# Overview of Optical Fiber Biosensors for the Detection of Cancer Biomarkers in the Past Decade

**DOI:** 10.1021/acsomega.6c00722

**Published:** 2026-04-02

**Authors:** Vikas, Akhilesh Kumar Mishra, Rajneesh Kumar Verma, Paola Saccomandi

**Affiliations:** † Department of Mechanical Engineering, 18981 Politecnico di Milano, Milan 20156, Italy; ‡ Department of Physics, 30112 Indian Institute of Technology Roorkee, Roorkee 247667, Uttarakhand, India; § Department of Physics, 30043 University of Allahabad, Prayagraj 211002, Uttar Pradesh, India

## Abstract

Early-stage detection of cancer plays a pivotal role in reducing mortality and improving patient survival. Consequently, extensive research has been directed toward the development of innovative diagnostic platforms that exploit cancer-specific biomarkers. These biomarkers encompass a diverse array of biological molecules including nucleic acids, proteins, enzymes, hormones, cytokeratins, and cell surface receptors that provide valuable insights into disease onset, progression, and therapeutic response. This review provides a comprehensive overview of recent progress in the design and application of optical fiber biosensors (OFBs) for the detection of cancer biomarkers. It systematically summarizes key cancer types and associated biomarkers, their physiological and pathological concentration ranges, and the corresponding sensing mechanisms utilized for their quantification. Emphasis is placed on the fundamental configurations of optical fiber biosensing systems, along with their working principles. Emerging trends and future research directions are also outlined to accelerate the development of next-generation OFBs for real-time and point-of-care cancer diagnostics.

## Graphical abstract

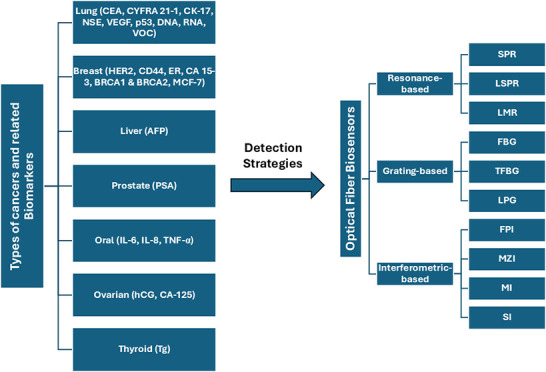

## 1 Introduction

Cancer remains the second leading cause of mortality worldwide, accounting for approximately 9.7 million deaths in 2022, according to the International Agency for Research on Cancer (IARC).^1^ It involves a diverse and complex group of diseases characterized by the loss of normal cellular growth regulation, uncontrolled proliferation, invasion of surrounding tissues, and eventual metastasis to distant organs.^2^ Its development is influenced by both endogenous and exogenous factors, including aging, radiation exposure, tobacco consumption, viral infections, metabolic disorders, and environmental pollutants.^3^ Despite significant advances in treatment and screening, cancer remains a leading global health challenge. The World Health Organization (WHO) projects that by 2050, the global number of new cancer cases will surpass 35 million annually, marking a 77% increase compared to 2022 estimates.^1^ Beyond its medical impact, cancer imposes considerable socioeconomic and public health burden.^4^

Early detection plays a critical role in improving survival outcomes. In this context, cancer-specific biomarkers have emerged as valuable tools for identifying malignancies at early stages, often prior to clinical symptom onset.^5,6^ The biomarker is a measurable biological indicator that reflects physiological or pathological processes within the body and includes a wide range of molecules such as nucleic acids, proteins, metabolites, enzymes, hormones, cytokeratin, and cell surface receptors.^7^ Biomarkers are detectable in blood, serum, saliva, urine, tissues, and other body fluids.^8^ Depending on their clinical application, biomarkers may be classified as diagnostic, prognostic, or predictive.^9^ Various types of cancer and related biomarkers are depicted in Figure 1. Alterations in the presence, concentration, or structure of these biomarkers often serve as early indicators of carcinogenesis.^10^

**Figure 1:**
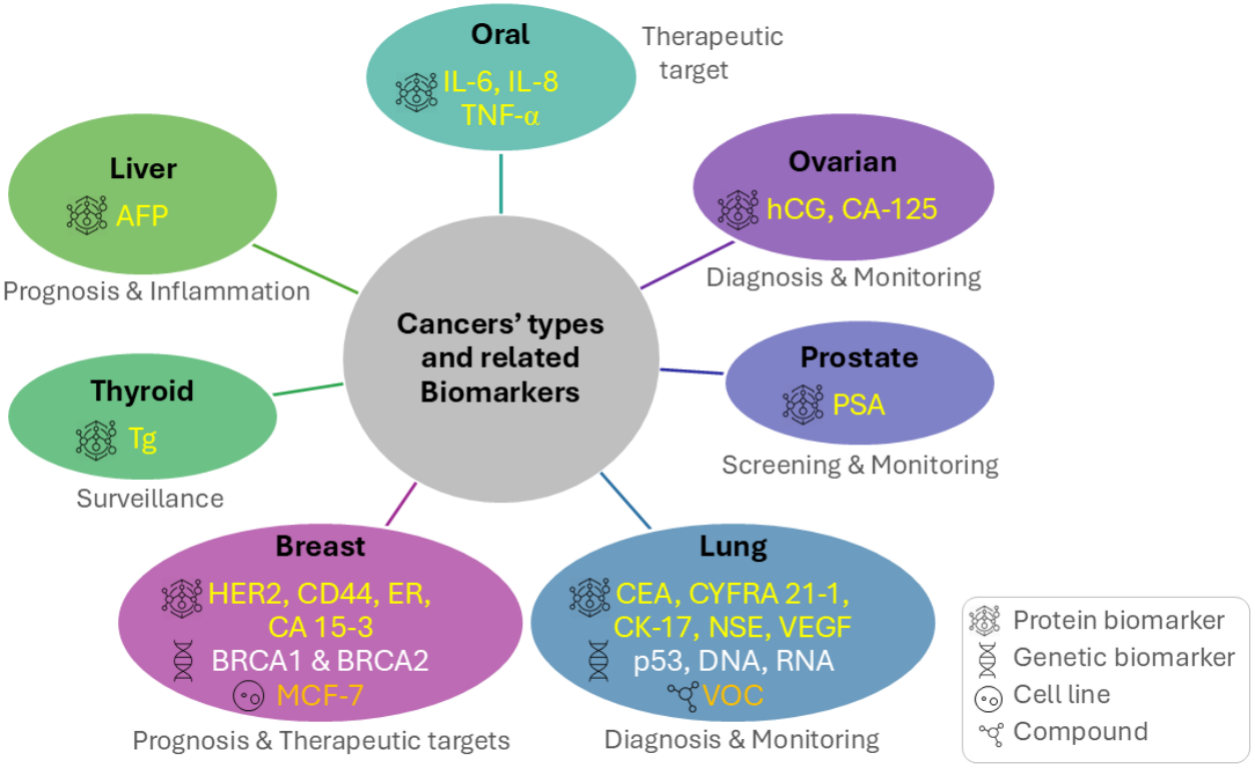
Schematic illustration of major cancer types and related biomarkers.

An ideal cancer biomarker screening assay should be rapid, cost-effective, easy to perform at the point of care, and capable of functioning in resource-limited settings. Currently, various assay-based techniques are employed for cancer biomarker detection, including enzyme-linked immunosorbent assay (ELISA), radioimmunoassay (RIA), Western blotting (WB), high-performance liquid chromatography (HPLC), polymerase chain reaction (PCR), immunohistochemistry (IHC), as well as imaging modalities such as computed tomography (CT), magnetic resonance imaging (MRI), and positron emission tomography (PET).^11–18^ Although these methods are well-established and highly robust, they have intrinsic limitations that reduce their suitability for early-stage, rapid, and routine clinical screening. For example, biochemical and molecular techniques offer high analytical reliability but typically require multiple processing steps, specialized reagents, and trained workers. Many of these methods are time-consuming, labor-intensive, and unsuitable for real-time monitoring, and some have high operational costs and safety concerns. Imaging techniques, while noninvasive and useful for functional assessment, are expensive, may require contrast agents or radiation exposure, and generally lack the molecular sensitivity necessary for early-stage tumor detection. These limitations highlight the need for innovative, simple, highly sensitive, and selective diagnostic approaches that are economically feasible and adaptable to point-of-care or low-resource settings.

In recent decades, biosensors have gained significant attention due to their high sensitivity, rapid response, accuracy, adaptability, reliability, and cost-effective fabrication, making them highly suitable for detecting tumor-associated biomarkers.^19,20^ They are designed to identify chemical or biological molecules, enabling continuous monitoring and assessment of health conditions. Biosensors can operate via electrochemical, optical, thermal, piezoelectric, magnetic, or micromechanical mechanisms, offering versatile approaches for precise and timely cancer detection. Among the various biosensing platforms, optical biosensors have emerged as one of the most powerful tools for cancer diagnostics, offering high sensitivity, real-time analysis, and multiplexing capability.^21^ These biosensors operate by measuring variations in optical properties, such as absorption, luminescence, fluorescence, polarization, transmission, or reflectance, that occur due to biochemical interactions between the analyte and recognition elements. A typical optical biosensor consists of three core components: a sensing layer, where selective interaction with the target occurs; an optical signal transduction unit, which converts biochemical reactions into measurable optical responses; and a signal processing or amplification system, which enhances and interprets the resulting signal. Optical biosensors have found broad applications across diverse domains such as food safety, environmental monitoring, biotechnology, and biomedicine, with the medical sector being the most dominant due to their ability to perform genetic analysis, protein quantification, and drug screening.^22,23^ In oncology, their role is particularly crucial, as they allow early, accurate, and noninvasive cancer detection. The integration of optical biosensing platforms with data analytics, artificial intelligence (AI), and machine learning (ML) algorithms has further enhanced their interpretative power, enabling real-time decision-making and predictive diagnostics for continuous patient monitoring and personalized treatment strategies.^24,25^

Within the spectrum of advanced optical technologies, optical fiber biosensors (OFBs) have emerged as particularly promising owing to their unique combination of features, i.e., compact size, high sensitivity, lightweight design, biocompatibility, immunity to electromagnetic (EM) interference, and suitability for remote or in situ sensing.^26–28^ Over the past decade, OFBs have evolved from laboratory-based platforms for biomarker detection and drug screening to real-time in vivo applications, facilitating minimally invasive cancer diagnosis and therapy monitoring.^29^ Their miniature and flexible nature also enables seamless integration into medical instruments such as catheters, endoscopes, and biopsy needles, significantly improving their clinical usability. Beyond cancer diagnostics, OFBs are widely applied in physiological monitoring, minimally invasive surgeries, biomechanical sensing, and the detection of infectious diseases and other pathological conditions.^30–33^

In this Perspective, we present recent advancements in OFBs developed over the past decade for the detection of cancer biomarkers. The paper is structured into three main sections to provide a comprehensive understanding of this rapidly evolving field. Section 2 outlines the fundamental classifications of OFBs configurations essential for cancer diagnosis with a concise description of their underlying working principles, including resonance-based, grating-based, and interferometric sensor architectures. Section 3 focuses on different types of cancer, their impact on human health, associated risk factors, key biomarkers, typical concentration ranges in healthy and diseased states, and corresponding detection approaches using OFBs. Section 4 summarizes the major findings and provides insights into the optimal design strategies for high-performance OFBs. Furthermore, future perspectives and future research directions are discussed. It is anticipated that this review will foster interdisciplinary interest and inspire innovative developments toward reliable, cost-effective, and clinically viable OFBs for early-stage cancer diagnosis. For readers seeking detailed information on specific OFBs structures such as tapered, U-shaped, D-shaped, microstructured fibers, etc., we refer to the recent comprehensive reviews on these geometries.^34,35^

## 2 Classification of Optical Fiber Biosensors

OFBs can be broadly divided into three principal categories, as illustrated in Figure 2. The first category includes resonance-based platforms, where thin films are deposited on the fiber surface; the second category consists of grating-based sensors, and the third category includes interferometric configurations that detect phase variations between multiple light paths within the fiber.

**Figure 2:**
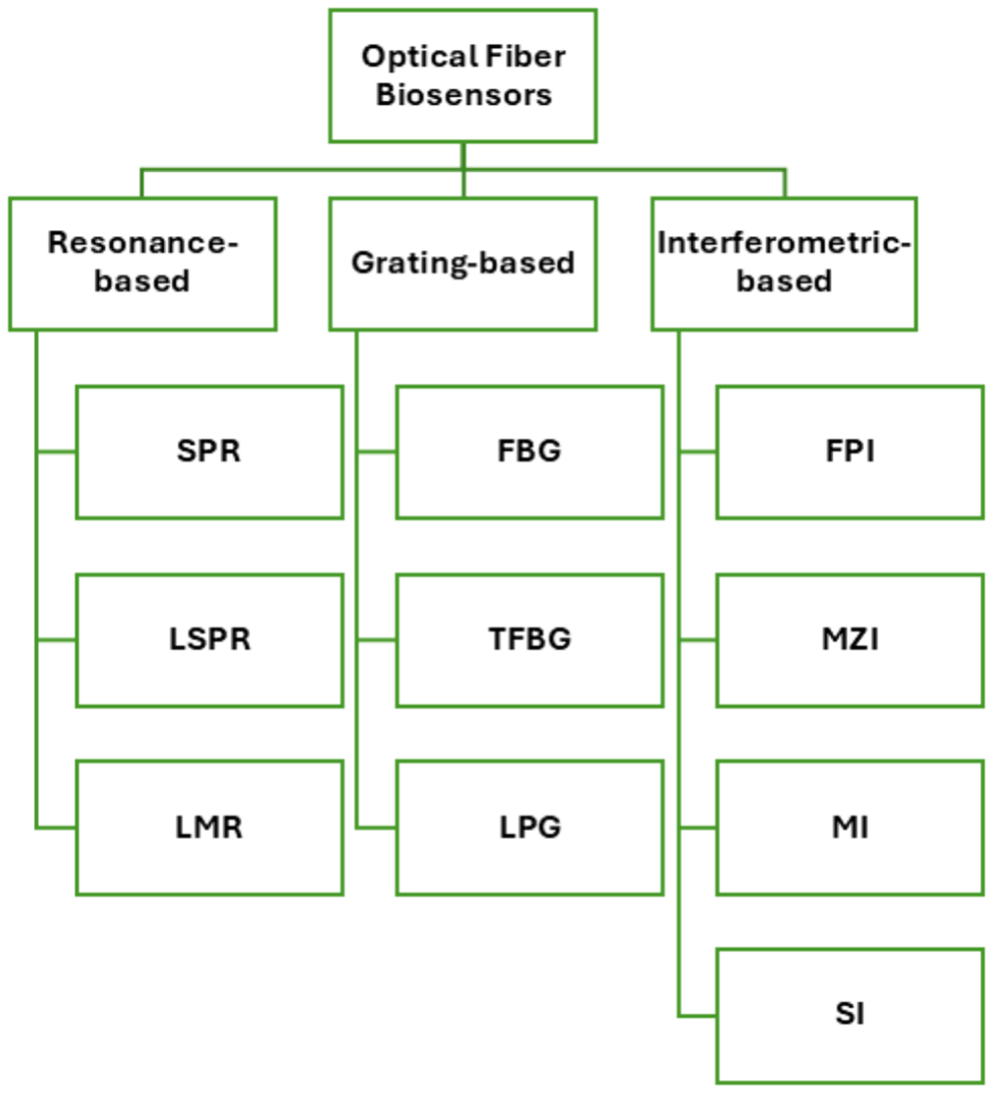
Portrayal of various OFB configurations analyzed in this review paper.

### 2.1 Resonance-Based OFBs

Resonance-based OFBs represent a prominent class of optical sensing technologies that utilize the resonant interaction between light and matter to achieve a high sensitivity and selectivity. Depending on the underlying resonance mechanism, these sensors are broadly categorized into three main types: surface plasmon resonance (SPR), localized SPR (LSPR), and lossy mode resonance (LMR). Each of these configurations operates on distinct sensing principles but shares a common objective: translating minute refractive index (RI) changes at or near the fiber surface into measurable optical signals. Despite their strong sensing performance, several challenges remain. Fabrication reproducibility is a key concern as variations in thin-film thickness, morphology, or nanoparticle distribution can affect resonance characteristics and lead to sensor-to-sensor variability. In complex biological samples, nonspecific adsorption and biofouling may cause spectral drift and reduce accuracy. Furthermore, large-scale clinical validation and standardized, cost-effective manufacturing strategies are still needed to ensure reliable translation of resonance-based OFBs from laboratory research to clinical application. The upcoming subsections provide a detailed discussion of the three principal resonance-based OFBs.

#### 2.1.1 Surface Plasmon Resonance (SPR)

The operation of an SPR-based OFB relies on the coupling between the evanescent field of guided light and surface plasmon waves (SPWs) at a metal–sample interface. When a p-polarized light propagates through the fiber core and reaches the core–metal boundary, attenuated total internal reflection (ATR) occurs. This process gives rise to an evanescent wave (EW) that penetrates the thin metal film and then the sensing medium, where it interacts with the SPW. When the propagation constants of both SPW and EW match, a resonance dip in the transmission spectrum is observed. This resonance condition can be expressed as^36^

1 $$\frac{\omega }{c}\sqrt{\varepsilon_{\mathrm{core}}}\sin\left(\theta \right)=\frac{\omega }{c}\sqrt{\frac{\varepsilon_{m}\varepsilon_{s}}{\varepsilon_{m}+\varepsilon_{s}}}$$

where the left and right terms represent the propagation constants of EW and SPW, respectively; ε_core_, ε_m_, and ε_s_ denote the dielectric permittivities of the fiber core, metal layer, and sensing medium, respectively. In practical implementation, a middle section of the cladding is typically removed to expose the fiber core, which is then coated with a thin metallic film.^37^ This coated region acts as the sensing zone, allowing a direct interaction between the guided light and the surrounding analyte. When the effective RI index of the external medium changes due to biomolecular interactions or environmental variations, the resonance condition also changes accordingly, causing a measurable displacement in the resonance wavelength (RW), toward either longer wavelengths (redshift) or shorter wavelengths (blueshift).

#### 2.1.2 Localized SPR (LSPR)

When light interacts with metallic nanoparticles (NPs) whose dimensions are comparable to the wavelength of the incident light, it induces coherent oscillations of the conduction electrons within the NP itself, known as localized surface plasmons (LSPs). Unlike SPR, where the oscillations are delocalized and propagate along a continuous metallic film, LSPs are confined to the surface of individual metallic NPs. Under the influence of the electric field, the conduction electrons within each NP are displaced relative to the positively charged atomic nuclei, creating a spatial charge separation. This displacement results in a restoring Coulombic force that drives the electrons back toward equilibrium, producing coherent oscillations of the electron cloud, thereby exciting the LSPR mode.^38,39^ The excitation of LSPs leads to a strong enhancement in the wavelength-dependent absorption and scattering of the EM field at the NP surface. This optical phenomenon can be quantitatively described using Mie theory, which provides analytical solutions to Maxwell’s equations under spherical boundary conditions to predict the optical extinction spectrum of metallic NPs. According to Mie theory, the extinction cross-section (*C*_ext_) for metallic NPs can be expressed by^40^

2 $$C_{\mathrm{ext}}=\frac{24\Pi^{2}NR^{3}{E_{s}}^{3/2}}{\lambda \ln\left(10\right)}\left[\frac{E_{\mathrm{imag}}}{{\left(E_{\mathrm{real}}+\chi E_{s}\right)}^{2}+{\left(E_{\mathrm{imag}}\right)}^{2}}\right]$$

In this equation, *R*/λ < 0.1, where *N* is the free-electron density, *R* is the radius of NP, and $E_{s}$ is the dielectric permittivity of the sensing medium. The term λ represents the incident wavelength, and ${E_{m}=E}_{\mathrm{real}}+iE_{\mathrm{imag}}$ is the complex dielectric permittivity of the metal. The parameter χ denotes the shape factor of the NP, which takes a value of 2 for spherical particles. In LSPR-based sensing, the absorption spectrum of light scattered or transmitted by metallic NPs is analyzed, and the position of the LSPR peak wavelength is extremely sensitive to the variations in the sensing medium RI (SMRI). Consequently, any interaction of biomolecules with the NP surface alters the local SMRI, which leads to a measurable shift in the LSPR wavelength. This forms the underlying principle of LSPR-based OFBs.^41,42^ The wavelength shift (Δλ) as a function of the SMRI change (Δ*n*_s_) is mathematically described by^43^

3 $$\Delta \lambda =S_{R}\left(\Delta n_{s}\right)\left[1-\exp\left(-\frac{2d}{L_{d}}\right)\right]$$

where *S*_R_ is the RI sensitivity, *d* denotes the effective thickness of the absorbed molecular layer, and *L*_d_ is the decay length of the associated EM field.

#### 2.1.3 Lossy Mode Resonance (LMR)

LMR occurs when a thin nanofilm having complex RI, typically a metal oxide, is coated onto an optical waveguide, leading to distinct attenuation bands in the transmitted light spectrum.^44^ This phenomenon results from the coupling between the guided modes of the optical waveguide and the lossy modes supported by the absorbing film.^45^ These lossy modes were historically referred to as leaky or long-range modes and theoretically analyzed using EM wave theory.^46^ In optical fiber configurations, LMR is achieved by depositing a thin absorbing film over the cladding-etched region of the fiber core and measuring the transmitted intensity at the output end. The first experimental and theoretical demonstration of LMR sensing was reported by Del Villar et al. in 2010 using indium tin oxide (ITO) films, which established the foundation for numerous subsequent studies.^47^ The origin of LMR lies in the phase-matching condition between the propagation constants of the waveguide and the lossy modes, where significant overlapping of mode fields occurs.^48^ Resonance arises when the real parts of these propagation constants are equal, and the lossy mode approaches its cutoff condition—determined by the incident light wavelength and the coating thickness. As a result, sharp dips appear in the transmission spectrum, corresponding to specific film thicknesses or wavelengths. The first LMR dip, located at longer wavelengths, typically exhibits the highest sensitivity, while additional LMR dips at shorter wavelengths (second, third, fourth order, etc.) can be generated by increasing the coating thickness and have lower sensitivities.^49,50^

### 2.2 Grating-Based OFBs

An optical fiber grating is a periodic modulation of the RI within the fiber core, first introduced by Ken Hill and colleagues in 1978.^51^ Fiber gratings are generally classified into two main types based on their periodicity: short-period gratings, with a period smaller than 100 μm, and long-period gratings, with periodic structures ranging from 100 μm to 1 mm.^52,53^ In grating-based OFBs, sensing performance is governed by precise grating inscription and subsequent surface functionalization. Small variations in the grating period, depth, or RI modulation can alter resonance characteristics and sensitivity. Additional variability may arise during the immobilization of biorecognition elements, influencing stability and batch-to-batch consistency. In biological samples, nonspecific adsorption and spectral cross-sensitivity, particularly in multiplexed grating arrays, can further complicate signal interpretation, necessitating optimized surface chemistry and selective binding strategies. For clinical translation, systematic validation using statistically significant patient cohorts is required to confirm the diagnostic accuracy under real-world conditions. Although grating inscription technologies are well established, standardized postinscription functionalization and scalable fabrication processes remain essential to ensure reproducibility, cost-effectiveness, and large-scale implementation. Depending on the design and fabrication method, fiber gratings can take several forms such as fiber Bragg gratings (FBGs), tilted FBGs (TFBGs), and long-period gratings (LPGs), as briefly explained in the upcoming subsections.

#### 2.2.1 Fiber Bragg Grating (FBG)

FBG is usually a single-mode fiber (SMF) that contains a periodic perturbation of the RI of fiber core along its length.^54^ This periodic structure of RI enables coupling from the forward propagating core mode to the backward counter-propagating core modes, producing a sharp reflection peak at a specific wavelength known as the Bragg wavelength λ_B_. A typical view of FBG and the corresponding spectrum is shown in Figure 3. The Bragg condition is given by^55^

4 $$\lambda_{B}=2n_{\mathrm{eff}}\Lambda$$

where *n*_eff_ is the effective RI of the core mode and Λ is the grating period. Only the wavelength satisfying this condition undergoes constructive interference, while the others are suppressed through destructive interference.

**Figure 3:**
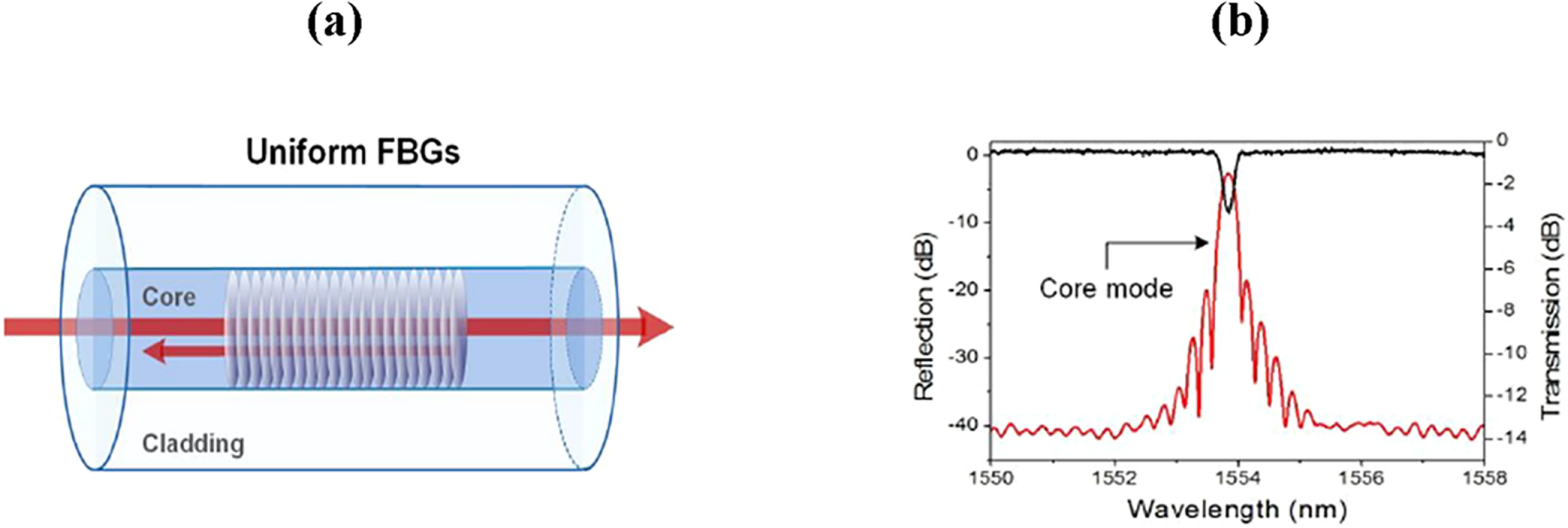
(a) Schematic illustration of light coupling in a typical FBG sensor and (b) example of transmitted (black) and reflected (red) spectra for a 1 cm long FBG sensor. Figures reprinted with permission under the terms of CC BY license from ref 56. Copyright 2017, MDPI.

FBGs are commonly fabricated using phase-mask technique, Talbot interferometer, and Lloyd mirror configuration, typically with femtosecond laser or ultraviolet laser sources.^57–59^ However, conventional FBGs are not inherently sensitive to the SMRI because the core is enclosed within a cladding of about 125 μm in diameter. This limits the interaction between the guided light and the external sensing medium. To enhance the interaction, the cladding can be etched to reduce its thickness, allowing the evanescent field to interact with the surrounding environment. Additionally, coating the etched fiber with a thin functional film can further increase the effective index and improve sensing performance, making FBGs more suitable for RI and biosensing applications.^60^ It should also be noted that conventional FBGs exhibit strong cross-sensitivity to temperature and strain, as variations in these parameters directly affect the Bragg wavelength. In biosensing applications, such environmental fluctuations may introduce spectral shifts unrelated to biomolecular interactions, thereby compromising measurement accuracy. Therefore, temperature and strain compensation strategies or reference configurations are often required to ensure reliable biochemical sensing.

#### 2.2.2 Tilted Fiber Bragg Grating (TFBG)

TFBGs are a special class of FBGs designed to enhance modal coupling and RI sensitivity. In these structures, the periodic RI modulation within the fiber core is tilted by an angle δ (typically <45°) relative to the fiber axis, breaking the cylindrical symmetry of the system.^61^ This tilt allows partial coupling of light from the core mode not only to the counter-propagating core mode, as in conventional FBGs, but also to various cladding modes (Figure 4). As a result, TFBGs exhibit multiple narrow attenuation bands in their transmission spectra, each corresponding to a distinct cladding mode. The RWs associated with these modes can be expressed as^62^

5 $$\lambda_{R\left(m\right)}^{TFBG}=\left(n_{eff}^{core}-n_{eff}^{clad\left(m\right)}\right)\frac{\Lambda }{\cos\left(\delta \right)}$$

where *n*_eff_^core^ and *n*_eff_^clad(*m*)^ are the effective RIs of the core and the *m*th cladding modes, respectively.

**Figure 4:**
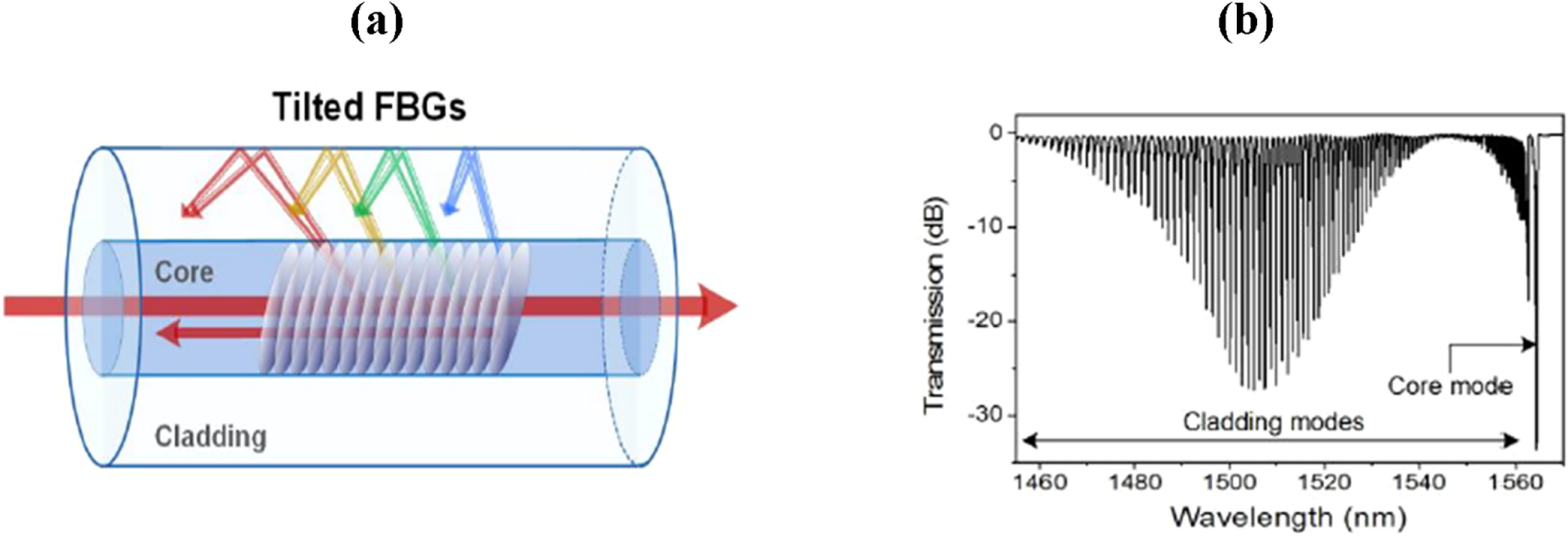
(a) Schematic representation of light coupling in TFBG sensor and (b) example of transmission spectrum for a 1 cm long TFBG with a 10° tilt angle. Figures reprinted with permission under the terms of CC BY license from ref 56. Copyright 2017, MDPI.

The spectral response of a TFBG depends strongly on grating parameters such as the tilt angle, grating period, length, and modulation strength.^63^ In addition, the effective RIs of the cladding modes are highly sensitive to changes in the SMRI. Therefore, as the SMRI increases, the RWs of the cladding modes shift toward longer wavelengths, providing a direct means of RI measurement. Moreover, similar to conventional FBGs, the performance of TFBGs is enhanced by using various strategies such as etching or thinning the fiber cladding, depositing thin functional films, or mismatching the cores between the input fiber and TFBG segment.^64^

#### 2.2.3 Long-Period Fiber Grating (LPG)

LPGs are optical structures characterized by a relatively long modulation period of the RI of the fiber core.^65^ LPG promotes coupling of light between the fundamental core mode and copropagating cladding modes, as shown in Figure 5. This coupling gives rise to multiple attenuation bands at various RWs, each associated with a specific cladding mode in the transmission spectrum. These RWs follow the phase-matching condition as given by^66^

6 $$\lambda_{R\left(m\right)}^{\mathrm{LPG}}=\left(n_{\mathrm{eff}}^{\mathrm{core}}-n_{\mathrm{eff}}^{c\mathrm{lad}\left(m\right)}\right)\Lambda$$

**Figure 5:**
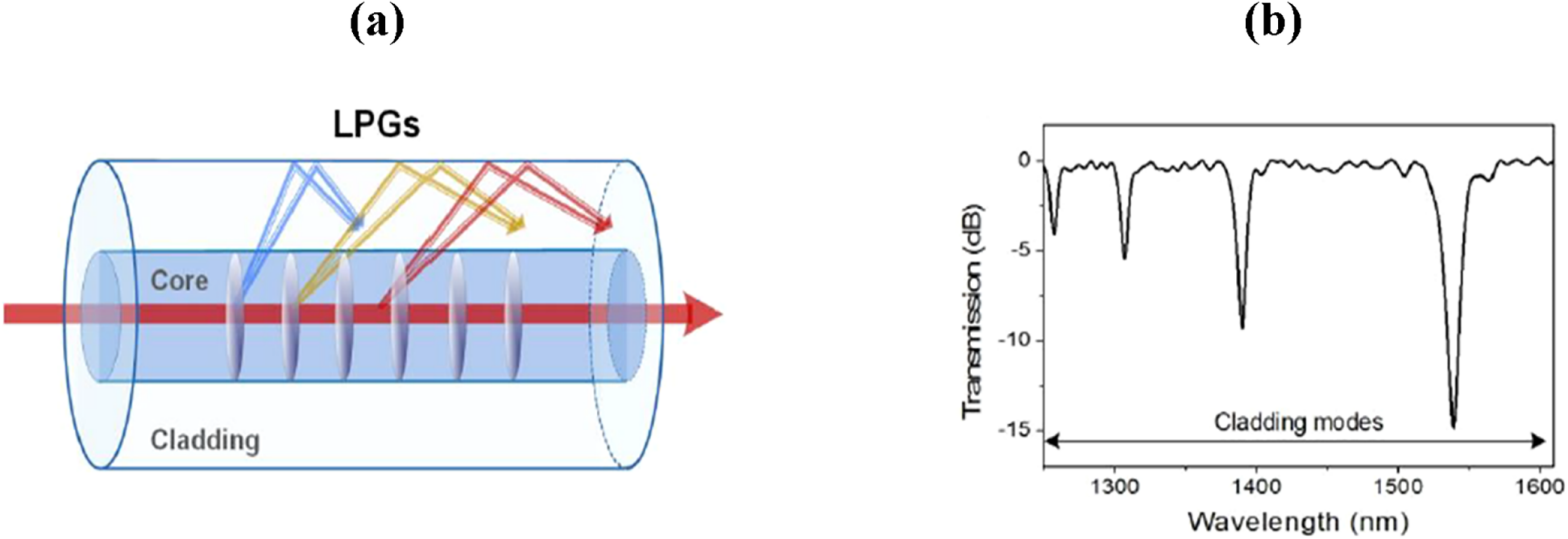
(a) Schematic diagram illustrating light coupling in an LPG sensor and (b) example of transmission spectrum for a 1 cm long LPG sensor. Figures reprinted with permission under the terms of CC BY license from ref 56. Copyright 2017, MDPI.

Since these cladding modes propagate near the cladding–medium interface, so any increase in SMRI causes a shift of the RW values.^67^ LPGs are commonly fabricated using amplitude masks, while simpler methods such as point-by-point inscription or periodic electric discharge can also be employed to tailor their grating characteristics.^68^

### 2.3 Interferometric-Based OFBs

In interferometric-based OFBs, incident light is typically split into two or more optical paths that propagate through different sections of the fiber and subsequently recombine. Due to variations in optical path lengths or RIs, the recombined light waves exhibit a relative phase shift, producing interference fringes that appear as alternating transmission and attenuation bands in the output spectrum. The resulting light intensity in the case of two-mode interference can be expressed as^69–71^

7 $$I=I_{1}+I_{2}+2\sqrt{I_{1}I_{2}}\cos\left(\phi \right)$$

where *I*_1_ and *I*_2_ represent the intensities of the individual light components, and ϕ = ϕ_1_–ϕ_2_ is the corresponding phase difference. When SMRI is varied, optical path lengths of the propagating beams are altered and lead to a corresponding change in their phase difference. This variation shifts the interference pattern observed in the output spectrum. In most interferometric OFBs, the sensing mechanism relies on RI variations caused by molecular binding events occurring on the fiber surface, where the magnitude of the shift is directly correlated to the analyte concentration. However, the specialty fiber-based interferometers are more valued for their unique structures and enhanced sensing capabilities.^72^ Fibers such as micro/nanostructured fibers (MNFs), microstructured optical fibers, photonic crystal fibers (PCFs), polymer fibers, hollow-core fibers (HCFs), dual-core fibers (DCFs), etc., enable highly sensitive measurements through features like air-hole designs, single-mode broadband operation, tunable dispersion, and strong birefringence.^73^ The subsequent subsections provide a detailed discussion of the four principal interferometric-based OFB configurations.

Despite their high phase sensitivity, interferometric OFBs require precise fabrication and alignment. Variations in the cavity length, taper geometry, or splicing conditions can affect interference stability and reproducibility. For multiplexed systems, accurate deconvolution of overlapping phase shifts is essential to maintain specificity. For clinical translation, standardized fabrication, robust packaging, and validation in large patient cohorts are necessary to ensure long-term stability and reproducibility. Additionally, simplifying device architectures and adopting scalable manufacturing strategies are crucial for cost-effective commercialization.

#### 2.3.1 Fabry–Perot Interferometer (FPI)

FPIs are generally classified into two types, i.e., extrinsic and intrinsic, based on how the reflective cavity is formed.^74^ In the extrinsic configuration, the cavity is created outside the fiber, typically as an air- or material-filled gap between two cleaved fiber ends supported by an external structure such as a capillary. In this case, the optical fiber mainly serves as a light-guiding medium, and any displacement or environmental change affecting the external reflector directly alters the interference signal. In contrast, intrinsic FPI (IFPI) has reflectors fabricated within the fiber itself, forming a glass microcavity that enables a more robust and integrated design. Although extrinsic FPIs (EFPI) are easier to fabricate, they often face alignment and coupling loss challenges, whereas intrinsic designs require advanced fabrication techniques such as femtosecond laser micromachining or chemical etching.^75^ In both cases, the device forms multiple beam interferences between parallel reflectors, and variations in parameters, such as temperature, strain, or RI, modify the optical path length, leading to a measurable spectral shift. The reflected intensity at the output of an FPI can be expressed by eq 7, in which the phase difference (ϕ) is given by^76^

8 $$\phi =\frac{4\pi \mathrm{nL}}{\lambda }$$

where *n* represents the RI of the cavity medium and *L* is the cavity length. By tracking shifts in the interference spectrum, FPIs enable precise measurement of various physical and chemical parameters. In cancer diagnostics, the cavity surface is typically functionalized with antibodies or nucleic acids, and biomarker binding alters the effective optical path length, producing measurable spectral variations. Owing to their high phase sensitivity and compact cavity architecture, FPI-based sensors are well suited for label-free and selective cancer biomarker detection. Although FPI sensors are widely used for temperature and strain sensing applications,^77^ their high phase sensitivity has also been adapted for refractive index-based biosensing relevant to cancer biomarker detection.

#### 2.3.2 Mach–Zehnder Interferometer (MZI)

In a typical MZI, the input light is divided into two independent arms (i.e., sensing arm and reference arm) and later recombined to form an interference pattern. Any change in the sensing arm’s length or the surrounding medium produces a phase difference, which can be measured to quantify the external parameters. MZI-based OFBs can be realized by using different techniques to generate interference between core and cladding modes. For instance, a thin-core fiber segment can guide part of the light in the core and part in the cladding before recombining in a standard SMF.^78^ Alternatively, core misalignment or the insertion of multimode fiber (MMF) segments can couple light into cladding modes, with the number of modes adjustable via fiber alignment.^79^ Moreover, tapered fiber sections also force core light into cladding modes and back, creating interference.^80^ In all the above cases, the arms may have the same physical length, and differences in effective RI indices between core and cladding modes result in distinct optical path lengths, with stronger evanescent fields in thinner fibers improving sensitivity. The output intensity for MZI sensors can be indicated by eq 7, in which *I*_1_ and *I*_2_ are the corresponding light intensities in the core and cladding, respectively, and the phase difference (ϕ) is written as^81^

9 $$\phi =\frac{2\pi \Delta n_{\mathrm{eff}}L}{\lambda }$$

where Δ*n*_eff_ represents the difference in effective indices between core and cladding modes and *L* is the fiber length between the splitter and combiner.

#### 2.3.3 Michaelson Interferometer (MI)

The MI operates similarly to an MZI, with the key difference that it relies on reflection rather than transmission, while maintaining comparable fabrication techniques and operating principles.^82^ In a typical MI configuration, light from an SMF is partially reflected at the junction between two fiber segments and again at the end surface of the reflective arm. The design can incorporate fiber segments with different core diameters, laterally offset splices, or a tapered fiber section inserted between two SMFs.^83–85^ Interference arises from the phase difference between the core mode and higher-order cladding modes, which depends on their effective RI difference (Δ*n*_eff_). The output intensity for MI can be indicated by eq 7, in which *I*_1_ and *I*_2_ are the corresponding light intensities in the core and higher-order cladding modes, respectively, and ϕ is the phase difference between the interfering modes. If *L* is the fiber length between the junction and the reflector, phase difference (ϕ) is defined by^86^

10 $$\phi =\frac{4\pi \Delta n_{\mathrm{eff}}L}{\lambda }$$

#### 2.3.4 Sagnac Interferometer (SI)

SI consists of a simple fiber loop where two light beams travel in opposite directions with distinct polarization states.^87^ An input beam is divided by a coupler, sending light through the loop in clockwise and counterclockwise directions before recombination at the same coupler. The optical path difference between the two beams arises from the variations in the propagation speeds caused by fiber birefringence. A polarization controller is used to adjust the polarization state, while the sensing section typically employs highly birefringent or polarization-maintaining fibers to enhance the polarization sensitivity. At the output end, interference occurs between the fast and slow polarization modes, resulting in a measurable phase difference (ϕ) which can be written as^88^

11 $$\phi =\frac{2\pi BL}{\lambda }$$

where *L* is the fiber loop length and *B* = |*n*_f_–*n*_s_| denotes the birefringence of the fiber, where *n*_f_ and *n*_s_ are the effective RIs of the fast and slow modes, respectively.

## 3 Types of Cancer and Their Detection Using OFBs

### 3.1 Lung Cancer

Lung cancer is one of the most prevalent malignancies and remains the leading cause of cancer-related deaths worldwide.^89^ It accounts for the highest mortality rate among all cancers, with approximately 1.8 million deaths reported globally in 2020.^3^ The overall five-year survival rate for lung cancer is the lowest compared to other major cancers (approximately 18%), while for breast, colorectal, and prostate cancers, it reaches around 90%, 65%, and 99%, respectively. This poor survival rate is primarily due to the disease being asymptomatic in its early stages, resulting in a delayed diagnosis when treatment becomes less effective. Although tobacco smoking is the primary risk factor and contributes to nearly 80% of cases, lung cancer can also develop in nonsmokers due to prolonged exposure to second-hand smoke, environmental pollutants, industrial carcinogens, and radiation.^90^ It is broadly classified into two major categories: small cell lung carcinoma (SCLC) and nonsmall cell lung carcinoma (NSCLC), with NSCLC accounting for nearly 80% of total cases.^91,92^ NSCLC is further subdivided into adenocarcinoma, squamous cell carcinoma, and large cell carcinoma, with each characterized by distinct cellular morphology and progression patterns. Conversely, SCLC represents about 15–20% of cases and is one of the most aggressive and rapidly proliferating forms of lung cancer. While SCLC tends to metastasize early and demonstrates an initial high response to chemotherapy and radiotherapy, most patients experience recurrence, leading to poor long-term outcomes.^93^ NSCLC, on the other hand, progresses more slowly but is often diagnosed at advanced stages, reducing the success rate of therapeutic interventions.^94^ The staging of NSCLC, ranging from stage I to IV, plays a crucial role in determining the appropriate treatment strategy and patient prognosis. In the early stages (I and II), the cancer is confined to the lungs and can be effectively managed with surgical resection, achieving a five-year survival rate of up to 75%.^95^ As the disease advances to stages III and IV, it spreads to nearby tissues and distant organs, drastically lowering survival prospects and complicating treatment options. Clinically, lung cancer symptoms such as chronic coughing, shortness of breath, chest pain, and weight loss are often nonspecific and may mimic other respiratory conditions, contributing to diagnostic delays.

#### 3.1.1 Biomarkers for Lung Cancer and Their Detection

Lung cancer biomarkers are mainly classified into protein-based and genetic/DNA-based categories, each offering distinct diagnostic advantages.^96,97^ Protein biomarkers mainly include carcinoembryonic antigen (CEA), cytokeratin family (CYFRA 21–1, CK-17, and CK-7), neuron-specific enolase (NSE), and vascular endothelial growth factor (VEGF).^98^ In parallel, genetic biomarkers including p53, DNA, RNA, and microRNAs have gained prominence due to their ability to reveal molecular alterations linked to oncogenesis and disease stage.^99^ These biomarkers are typically detected in biological fluids, such as blood, sputum, bronchial lavage, or nasal epithelium. More recently, attention has also shifted toward volatile organic compounds (VOCs)-based biomarkers detected in exhaled breath, which provide a promising route for rapid, noninvasive, and real-time screening of lung cancer.^100^ The subsequent subsections present a detailed discussion of these biomarker categories, emphasizing their clinical significance, detection strategies, and integration into OFBs for lung cancer diagnosis.

##### 3.1.1.1 Carcinoembryonic Antigen (CEA)

CEA is among the most extensively studied protein biomarkers for lung cancer.^101^ It is a glycoprotein involved in cell adhesion and is normally expressed during fetal development with minimal production in healthy adult tissues.^102^ In healthy individuals, the serum CEA concentration typically remains below 5 μg/L, whereas levels exceeding 20 μg/L are often associated with the presence of lung cancer.^103^ Notably, smokers tend to exhibit slightly higher baseline CEA levels compared to nonsmokers. Several studies have also reported a significant correlation between increased serum CEA concentrations and reduced survival time in patients with NSCLC.^104^

To address the need for reliable CEA detection, an optical microfiber coupler (OMC)-based biosensor was reported for quantifying CEA in human serum.^105^ The sensor effectively tackled nonspecific adsorption and variability among clinical samples through a serum preadsorption technique, which established a stable antifouling interface on the OMC surface. The OMC biosensor demonstrated a detection limit of 34.6 fg/mL and maintained good sensitivity in complex serum environments. Moreover, the correction strategy using 30% Sigma HS reduced individual variability to within 2–7.5% of the linear range, yielding results consistent with standard clinical tests. Another promising approach was based on FPI integrated with the Vernier effect for CEA detection.^106^ The FPI was fabricated by splicing two SMFs with a lateral offset, creating sensing and reference cavities that together produce dual interference fringes. The resulting Vernier effect amplified the RI sensitivity by nearly seven times, achieving −8535 nm/RIU compared with 1174 nm/RIU in the single-cavity configuration. Upon functionalization with anti-CEA antibodies, the sensor exhibited highly specific CEA detection, with a detection limit of 36.14 fg/mL and a rapid response time under 30 min. In addition, a TFBG-based hybrid plasmonic biosensor was developed to enhance CEA sensing performance in both buffer and serum environments.^107^ The system integrated polyacrylonitrile (PAN) nanofibers, functionalized with covalently attached anti-CEA antibodies with a gold (Au) nanomembrane, as shown in Figure 6(a). The synergistic effect of the TFBG and SPR enhanced the optical signal, achieving a sensitivity of 0.46 dB (μg/mL)^−1^ and a detection limit of 505.4 ng/mL (Figure 6(b–d)).

**Figure 6:**
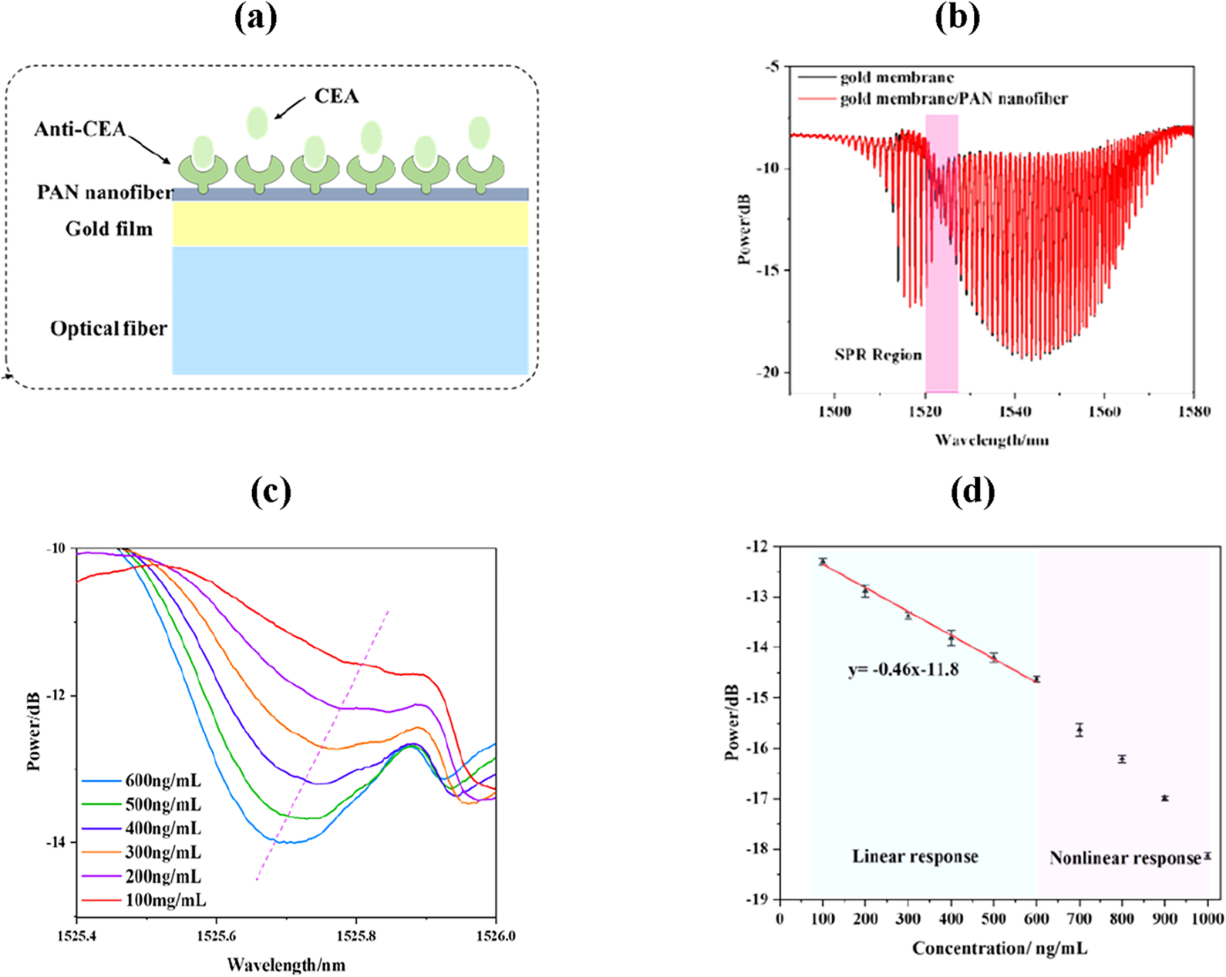
(a) Schematic illustration of the TFBG-based plasmonic biosensing probe. (b) SPR region of sensor coated with Au layer and with Au/PAN composite layer. (c) Transmitted curves for different CEA concentrations and (d) sensitivity analysis. Figures reprinted with permission under the terms of the Optica Open Access Publishing Agreement from ref 107. Copyright 2024, Optica Publishing Group.

A recent advancement involves the integration of specklegram analysis and two-dimensional convolutional neural networks (2D-CNNs) for the detection of CEA.^108^ The proposed biosensor utilized a tapered MMF with a waist diameter of 7.46 μm, enhancing the evanescent field for efficient interaction with CEA analytes. Functionalization with CEA antibodies allowed selective binding, while the resulting interference-induced speckle patterns vary with a change in RI. Experimental results indicated a surface sensitivity of 0.0012 (ng/mL)^−1^ and a limit of detection (LOD) of 1 ng/mL within the 1–50 ng/mL concentration range. To overcome nonlinear response behavior at higher concentrations, a 2D-CNN model was trained to interpret the specklegrams, achieving a maximum detection error of just 0.358%. Moreover, a molybdenum disulfide (MoS_2_)-functionalized microfiber interferometer has demonstrated remarkable potential for CEA detection.^109^ The biosensor achieved a detection limit of 15.86 fg/mL, an RI sensitivity of 1915.9 nm/RIU, and a response time of less than 15 min.

##### 3.1.1.2 CYFRA 21–1, CK-17, and CK-7

CYFRA 21–1 is the smallest member of the human cytokeratin family with a molecular weight of approximately 40 kDa and is recognized as an important biomarker for NSCLC, particularly squamous cell carcinoma.^110^ Enhanced serum concentrations of CYFRA 21–1 are commonly observed in lung cancer patients, with a reported diagnostic specificity of about 87% at a threshold level of 3.6 ng/mL.^111^ Beyond its diagnostic relevance, CYFRA 21–1 levels have been shown to correlate strongly with disease progression and overall clinical status of lung cancer patients, often demonstrating a stronger association than CEA or NSE.^112^ CK-7 and CK-17 have also been extensively studied, with CK-7 frequently detected either as a full protein (∼78 kDa) or as smaller peptide fragments (∼2.6 kDa) and CK-17 serving as another biomarker for both serum- and tissue-based diagnostics.

In 2016, Ribaut and co-workers constructed a surface plasmon resonance- tilted fiber Bragg grating (SPR-TFBG) sensor by exploiting RI change near the Au-coated fiber surface to monitor molecular binding.^113^ Comparative studies showed that this sensor could provide high sensitivity for both the full CK-7 protein and smaller peptide fragments, with a detection limit reaching up to 0.4 nM. The incorporation of optimized antibody–antigen cross-linking further ensured selectivity and minimal interference from surrounding media. Further studies comparing sputtering and electroless plating methods have highlighted that both the thickness and surface roughness of the Au layer critically affect the cladding mode distribution and, consequently, the SPR-TFBG sensor’s sensitivity.^114^ Optimized configurations have achieved a detection limit as low as 14 fM for CK-17. Beyond plasmonic enhancement, bare TFBGs without metallic coatings have also shown promise for lung cancer biomarker detection.^115^ Antibodies immobilized directly onto the silica fiber surface leverage the intrinsic sensitivity of cladding modes near their cutoff regions. Among functionalization strategies, electrostatic adsorption was found to be the most rapid and effective, achieving a detection limit of 14 pM for CK-17 detection. Most recently, Qiu’s group proposed the detection of CYFRA 21–1 through highly sensitive OFB based on cascaded dual FPIs combined with the high-order harmonic Vernier effect (HVE).^116^ In that study, SMF and hollow-core PCF formed the dual-FPI structure, while graphene oxide (GO) coatings increased the functional surface area for antibody immobilization. The HVE significantly enhanced RI sensitivity to 162,000 nm/RIU with LOD as 1.6 fg/mL.

##### 3.1.1.3 Neuron-Specific Enolase (NSE)

NSE is a 78 kDa glycolytic enzyme predominantly expressed in mature neurons, where it catalyzes the conversion of 2-phospho-d-glycerate to phosphoenolpyruvate and water during glycolysis.^117^ Its enzymatic activity is calcium-dependent and requires magnesium ions as cofactors for catalytic efficiency and dimer stabilization. Under physiological conditions, serum NSE levels are typically low; however, concentrations exceeding 9 ng/mL are frequently observed in patients with lung cancer, particularly in SCLC.^118^ NSE has emerged as one of the most reliable diagnostic biomarkers for SCLC, with overexpression reported in approximately 70% of cases and elevated levels in up to 90% of patients with advanced lung cancer stages.^119^ Although NSE alone provides sufficient diagnostic value for SCLC, its combination with CEA and CYFRA 21–1 enhances diagnostic accuracy and prognostic assessment, particularly for distinguishing NSCLC from SCLC.^120^ Notably, Zhou et al. introduced a novel black phosphorus (BP)-based OFB for NSE detection.^121^ The sensor integrated BP nanosheets with a largely TFBG, with poly-l-lysine serving as a cross-linker to establish a robust bionanophotonic interface. Anti-NSE antibodies immobilized on the BP-TFBG enabled specific binding, achieving an LOD of 1.0 pg/mL, which is 4 orders of magnitude below the clinical cutoff for SCLC.

##### 3.1.1.4 Vascular Endothelial Growth Factor (VEGF)

VEGF is a homodimeric glycoprotein that plays a central role in angiogenesis and vascular permeability.^122^ The typical concentration range of VEGF in serum is found to be 256 pg/mL in healthy individuals to around 434 pg/mL in cancer patients.^123^ Increased VEGF expression in tumors is often associated with activation of the angiopoietin/Tie signaling pathway, which contributes to the disruption of vascular integrity and promotes the formation of aberrant, leaky blood vessels within the tumor microenvironment.^124^ In this context, an SPR-based biosensor utilizing a plastic optical fiber (POF) was designed for detecting VEGF.^125^ The sensing platform integrates two essential components: a functional layer composed of DNA aptamers (short oligonucleotides capable of binding specific non-nucleic-acid targets with high affinity and selectivity) and a planar Au-coated POF structure that provides an efficient interface for biomolecular recognition. This configuration achieved the lowest detection limit of 3 nM for effective VEGF detection.

##### 3.1.1.5 p53

In normal lung tissue, p53 protein is typically undetectable due to its very short half-life.^126^ However, mutations in the p53 gene increase the stability of the protein, leading to its accumulation in cells at levels that can be detected using OFBs. Mutations in the p53 gene are reported in approximately half of NSCLCs with prevalence ranging from 34% to 82% depending on the study and patient cohort. For instance, Cheng et al. observed abnormal p53 expression in nearly 58% of NSCLC patients.^127^ Furthermore, p53 expression patterns vary according to histological subtypes of lung cancer, reflecting differences in molecular pathology and clinical behavior.^128^ To achieve rapid and precise p53 detection, an interferometric-based cascaded seven-core OFB was utilized.^129^ This device enabled sensitive detection of p53 protein over a concentration range of 0.05–5 ng/mL, with LOD noticed to be 0.0496 ng/mL. Comparative analysis using envelope and single-trough methods showed that the envelope-based approach provided about 8.37 times higher sensitivity (i.e., 3.507 nm/log­(ng/mL)) than the single-trough method. Further optimization revealed that increasing the antibody concentration from 32 to 100 μg/mL enhanced wavelength shifts at higher p53 concentrations. This improvement was attributed to greater antibody immobilization, which improved the protein binding efficiency. Importantly, when tested using 293T cell lysates, the designed OFB achieved a much lower LOD compared with the conventional WB technique.

##### 3.1.1.6 DNA, RNA, and MicroRNAs

Circulating nucleic acids, including DNA and RNA fragments, have emerged as promising biomarkers for lung cancer detection.^130^ Among these, mRNAs (mRNAs) and microRNAs (miRNAs) are of particular interest due to their regulatory roles in gene expression. miRNAs are short RNA molecules, typically 19–25 nucleotides in length, that regulate gene expression by binding to target mRNAs and either promoting their degradation or inhibiting their translation.^131^ In lung cancer, both upregulation and downregulation of specific miRNAs have been documented. For instance, elevated expression of miR-21 in NSCLC patients distinguished them from healthy individuals with 100% specificity and 70% sensitivity.^132^ Other miRNAs have demonstrated similar diagnostic performance, with specificity ranging from 92% to 100% and sensitivity between 75% and 85%.^133^

SPR-based configurations primarily relied on direct hybridization approaches, achieving nanomolar-level detection limits in buffer solutions.^134–136^ Subsequent incorporation of 2D materials and nanostructured plasmonic interfaces reduced detection limits into the picomolar regime.^137,138^ To address the ultralow abundance of miRNAs, enzymatic and catalytic amplification strategies, including DNA walker mechanisms, rolling circle amplification (RCA), and G-quadruplex-assisted signal enhancement, enabled femtomolar and attomolar sensitivity in biological fluids.^139–147^ More recent platforms demonstrate a clear transition toward portable architectures and direct analysis in serum, saliva, and sweat samples.^148,149^ A summary of OFB-based nucleic acid detection strategies is provided in Table 1.

**Table 1:** OFB-Based Strategies for the Detection of DNA, RNA, and MicroRNAs (miRNAs) That Are Relevant to Lung Cancer^a^

fiber configuration	detection mechanism	amplification strategy	target	sample	linear range	LOD	key advancement	refs
Au-coated fiber	SPR	Direct hybridization	exon-20 DNA	Buffer	nM range	9 nmol/L	Dual DNA and temperature sensing	^134^
MoSe_2_-coated fiber	LSPR	2D material enhancement	cDNA	Buffer	0.07–1000 nM	88.58 pM	Rapid response(1s)	^137^
AuNRs-functionalized fiber	LSPR	Nanorod enhancement	RNA	Buffer	1 pM–50 nM	1 pM	Dual-parameter detection	^138^
AuNPs-modified fiber	Plasmonic	DNA walker + RCA	miRNAs	Serum	fM range	8.32–107.15 fM	Dual-mode enzymatic amplification	^145^
Optical microfiber + GO	Plasmonic	Bimetallic nanorods	miR-21	Serum/Sweat	amol range	0.5–0.9 aM	Portable and intracellular detection	^148^
Au@ZIF-8 + TFBG	Plasmonic + TFBG	Core–shell nanostructure	miRNA	Serum/Saliva	fM range	7.78–13.6 aM	Ultrasensitive detection in biofluids	^149^

a MoSe_2_: molybdenum diselenide; AuNRs: gold nanorods; AuNPs: gold nanoparticles; GO: graphene oxide; ZIF: Zeolitic Imidazolate Framework-8.

##### 3.1.1.7 Volatile Organic Compounds (VOCs)

Human exhaled breath contains a complex mixture of VOCs, which can originate from metabolic activity within the body or from external environmental sources.^150^ Recently, analyzing VOCs in breath has gained attention as a noninvasive method for detecting lung cancer.^151^ Common VOC biomarkers include acetone, ethanol, methanol, isopropanol, benzene, methylbenzene, chloroform, toluene, etc.^152,153^ Differences in the VOC composition between healthy and affected individuals highlight the potential of breath analysis as a diagnostic tool for early detection of lung cancer.

In this direction, Hromadka’s group demonstrated an LPG sensor functionalized with mesoporous films infused with calixarenes (CA[4] and CA[8]) that interact selectively with VOC molecules.^154^ Alternating layers of SiO_2_ nanoparticles and poly­(allylamine hydrochloride) provided a high surface area for VOC adsorption, while operation at the phase-matching turning point enhanced the RI sensitivity. When tested with chloroform, benzene, toluene, and acetone vapors, the sensor achieved rapid responses within 30 s and displayed distinct spectral patterns for different VOC mixtures. The CA[4]-based configuration yielded the highest response, confirming that molecular cavity size influences interaction strength and sensitivity. Further advancement was achieved using zeolitic imidazolate frameworks (ZIFs), which combine structural stability with selective adsorption capability. A ZIF-8-coated LPG sensor detected acetone and ethanol with sensitivities of 0.015 ± 0.001 nm/ppm and 0.018 ± 0.0015 nm/ppm, and detection limits of 6.67 and 5.56 ppm, respectively.^155^ In a similar work, Wu et al. introduced a zeolite-coated spherical-end OFB forming an in-line FPI cavity for VOC detection.^156^ Adsorption of gas molecules on the zeolite surface induced wavelength shifts in the interference spectrum, enabling selective and sensitive detection. The sensor showed remarkable response toward isopropanol (281.9 pm/ppm) and comparatively weaker sensitivity to formaldehyde (4.99 pm/ppm). Even in mixed-gas environments, the fabricated sensor maintained precise isopropyl alcohol detection with minimal interference. Sensor response was further enhanced by fabricating a 32 μm FPI cavity using photopolymerizable resin at the fiber end.^157^ The resulting sensor exhibited distinct temporal and spectral responses to methanol, ethanol, and isopropanol vapors with detection limits down to 1 ppb. Moreover, Wang et al. functionalized a side-polished silicon wafer with ZIF-8 to form an open-cavity FPI coupled with a fiber patch cable and 3D-printed structure.^158^ The sensor showed sensitive detection of methylbenzene, methanol, and ethanol, with sensitivities of 0.118, 0.177, and 0.412 pm/ppm, respectively.

Metal organic frameworks (MOFs) have been successfully incorporated in OFBs for VOC detection. In this context, an LSPR sensor was developed by immobilizing AuNPs on the fiber tip, followed by HKUST-1 MOF functionalization using a layer-by-layer approach.^159^ The number of MOF coating cycles (40–120) influenced adsorption dynamics, with thicker layers enhancing VOC sensitivity. Sensors with 80 and 120 cycles exhibited red-shifts in RW, reaching sensitivities up to 15.5 nm/% for ethanol and a detection limit of 0.003%. Parallel to MOF-based developments, 2D nanomaterials have also been coated over the OFB surface to enhance surface interaction with VOC molecules. For example, Jamila et al. synthesized porous boron nitride nanosheets (BNNSs) and coated them on cladding-modified optical fibers for detecting ammonia, ethanol, and acetone in the 0–300 ppm range.^160^ The sensor exhibited high selectivity for ammonia, achieving a 55% response at 300 ppm with rapid response and recovery times of 15 and 34 s, and a detection limit of 13.5 ppm. The improved sensing behavior was attributed to the porous BNNS architecture that promoted efficient charge transfer and Fermi-level modulation upon gas adsorption.

Recently, a poly­(dimethylsiloxane) (PDMS)-based OFB was fabricated using intermode interference between two MMFs coupled by a stretched PDMS fiber.^161^ The adsorption of VOC molecules caused changes in the RI and volume of the PDMS medium, leading to measurable wavelength shifts. The sensor detected acetone, ethanol, methanol, toluene, and ether vapors with sensitivities of 4.39 to 14.84 pm/ppm over 0–400 ppm, while offering mechanical robustness, humidity resistance, and facile fabrication. Complementing this, Shao et al. developed a heterogeneous multimode fiber–single-mode fiber–multimode fiber (MMF–SMF–MMF) sensor coated with chromium and gold films, with a PDMS overlayer acting as the VOC-responsive element.^162^ For more selective isopropanol detection, Pathak et al. introduced a molecularly imprinted polymer (MIP) on an Au-coated optical fiber.^163^ The resulting Au/MIP sensor achieved a 7-fold higher sensitivity (0.014 nm/ppm) than the bare MIP-coated fiber and had a detection limit of 82.41 ppm.

### 3.2 Breast Cancer

Breast cancer is the most prevalent malignant disease affecting women across the globe and ranks second in mortality after lung cancer.^164^ In 2018 alone, around 627,000 women lost their lives to breast cancer, representing roughly 15% of all female cancer-related deaths.^165^ This involves a heterogeneous group of malignancies that commonly originate in the epithelial cells of the milk ducts or lobules, known as ductal and lobular carcinomas, respectively.^166^ The onset of breast cancer is influenced by multiple factors, including age, reproductive history, inadequate breastfeeding, heredity, lifestyle, and geography.^167^ Behavioral factors such as alcohol intake, tobacco use, physical inactivity, and obesity further elevate the risk. Age-related reproductive patterns play a significant role in tumor development; women with a prolonged reproductive phase are more likely to develop breast cancer.^168^ Moreover, early childbirth reduces this risk; i.e., women who deliver their first child before age 20 are about half as likely to develop breast cancer compared to those who give birth after age 30. The likelihood increases even further when the first pregnancy occurs after age 35.^168,169^

#### 3.2.1 Biomarkers for Breast Cancer and Their Detection

Potential biomarkers for detecting breast cancer include human epidermal growth factor receptor 2 (HER2), cluster of differentiation 44 (CD44), estrogen receptor (ER), BRCA1/BRCA2 genes, cancer antigen 15–3 (CA 15–3) protein, and Michigan Cancer Foundation 7 (MCF-7). The upcoming subsections present a detailed discussion of these biomarkers and their detection strategies based on OFBs.

##### 3.2.1.1 Human Epidermal Growth Factor Receptor 2 (HER2)

HER2 is a critical biomarker in breast cancer, with its expression closely linked to both disease onset and progression. It is a transmembrane receptor protein involved in the regulation of normal breast cell growth, repair, and differentiation.^170^ However, the overexpression of HER2 promotes uncontrolled cellular proliferation and leads to tumor development. Approximately 20–30% of breast cancer cases exhibit HER2 overexpression.^171^ The physiological concentration of HER2 in healthy individuals typically ranges from 2 to 15 ng/mL, while HER2­(+) breast cancer patients may exhibit levels as high as 75 ng/mL, often associated with more aggressive tumor phenotypes compared to HER2(−).^172,173^

Sun et al. experimentally designed an OFB that detects HER2 using a taper fiber interferometer embedded in a FBG system.^174^ The experimental setup of the proposed sensors is shown in Figure 7(a). Moreover, as displayed in Figure 7(b), the fabricated FBG was insensitive to the change SMRI but was highly sensitive to the temperature change from 30 to 90 °C. On the other hand, the tapered fiber interferometer was highly sensitive toward the SMRI with a sensitivity of 2333 nm/RIU. When the sensor was characterized to sense HER2 (concentration 10 nm/mL) in real time for 60 min, a rapid change in wavelength (around 1.10 nm) was detected during the first 10 min of immersion, as depicted in Figure 7(c,d). It was also concluded that the proposed sensor functionalized with HER2 antibodies could provide an LOD of 2 ng/mL for the detection of HER2 biomarker.

**Figure 7:**
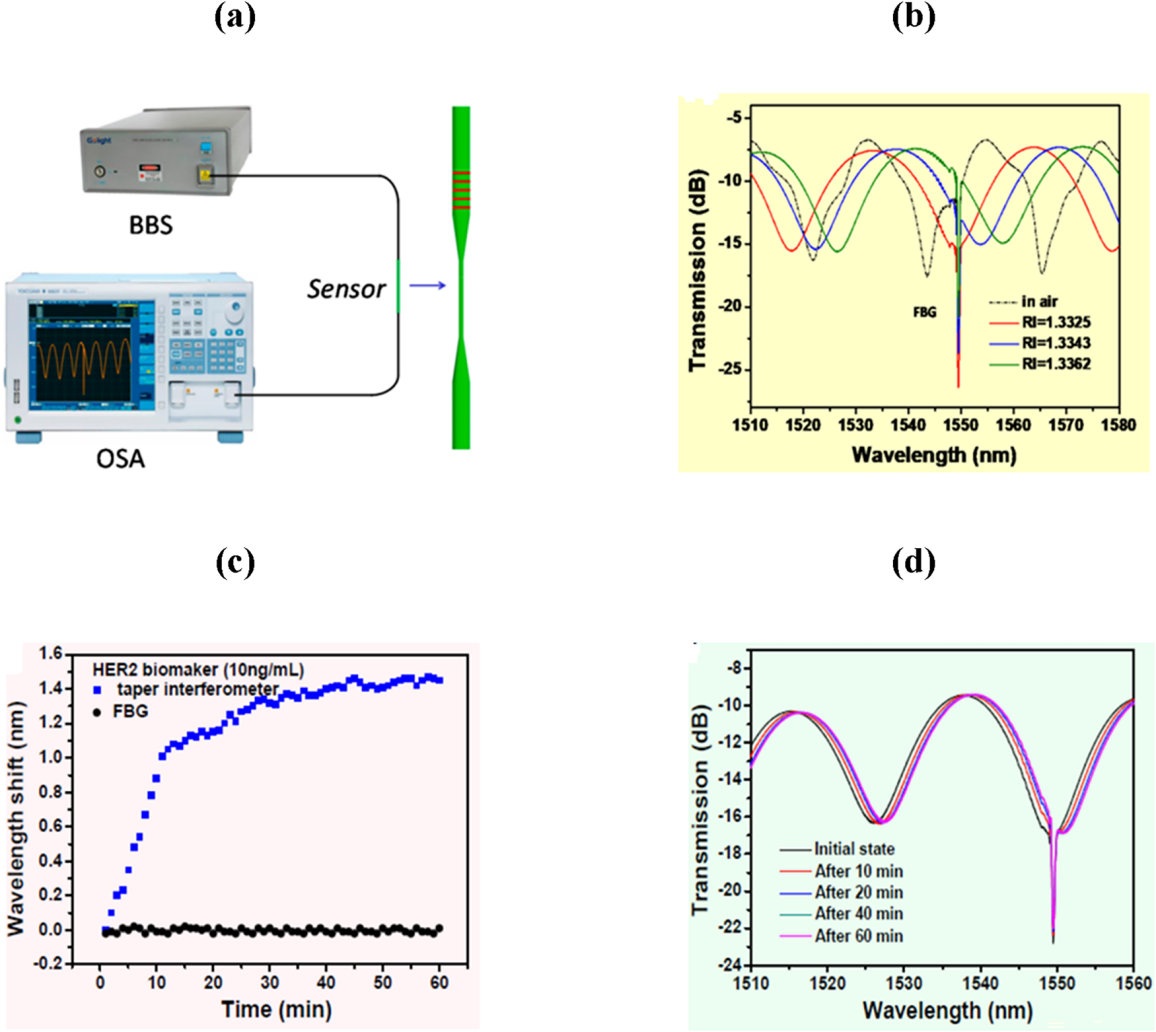
(a) Experimental sensing setup (Photograph courtesy of Sun, D.; Ran, Y.; Wang, G., Copyright 2017, MDPI). (b) Transmission curves of designed sensor for various SMRIs. (c) Real-time wavelength measurement of developed sensor for each taper interferometer and FBG sensor. (d) Variation in transmission curves at different time intervals. Figures reprinted with permission under the terms of CC BY license from ref 174. Copyright 2017, MDPI.

Building on this approach, a plasmonic-based TFBG biosensor was fabricated for HER2 monitoring using a demodulation system.^175^ First, thiolated aptamers were immobilized on the sensing surface, and then anti-HER2 antibodies were conjugated to enhance the sensor performance. Next, the functionalized sensor was dipped in HER2 sample with a concentration of 10^–6^ g/mL, and a redshift of around 800 pm was measured in surface plasmon resonance-tilted fiber Bragg grating (SPR-TFBG) spectrum, which is 2.5 times higher than the sensor without antibodies. Similar work was done to sense the HER2 in which the authors used an Au-coated TFBG sensor functionalized with anti-HER2 aptamer.^176^ The reported sensor exhibited an increase in wavelength shift for HER2 biomarker in the concentration ranging from 10^–12^ to 10^–6^ g/mL. Similarly, partially Au-coated TFBG sensor was effectively used for the detection of HER2 biomarker in phosphate-buffered saline (PBS) with different concentrations.^177^

Further innovations include the label-free detection of the HER2 biomarker in both PBS and serum using silica microfiber interferometry in the concentration range of 0–100 ng/mL.^178^ The sensing surface of silica-based microfiber was grafted with HER2 antibodies, resulting in a sensitivity of 0.1 nm/(ng/mL) and LOD of 0.5 ng/mL toward the HER2 biomarker. Another label-free SPR-based OFB was proposed to specifically detect HER2 biomarker in PBS.^179^ For this purpose, an MMF with a 400 μm core diameter was considered and coated with Au-film, which was further modified with anti-HER2 ssDNA aptamers. The developed SPR sensor was tested with 1 μg/mL concentration of HER2 protein and resulted in a sensitivity of 0.17 nm/nM and the lowest LOD of 9.3 × 10^–9^ g/mL. Later, Sypabekova et al. developed an OFB for detecting soluble HER2 (sHER2) biomarker in serum and PBS by splicing TFBG with a ball-tip resonator.^180^ For this, a ball resonator with 585 μm ball diameter was fabricated using a CO_2_ laser splicer at the tip of an SMF, as shown in Figure 8(a). The surface of the TFBG-ball resonator was then functionalized with Trastuzumab, an anti-HER2 monoclonal antibody that acts as a receptor to recognize sHER2. Figure 8(b) displays the sensing setup used for the experiment in which the amplitude response was recorded by using an interrogator. The developed sensor was tested in both PBS and 1/10 diluted serum for different concentrations of sHER2 up to 128 ng/mL, with corresponding LOD values being 151.5 ag/mL and 3.716 pg/mL, respectively. But it is worth noting that the change in amplitude for sHER2 in PBS was toward higher wavelengths, while the amplitude in serum had a decreasing trend. The calibration curve for detecting sHER2 in serum is shown in Figure 8(c), which shows a strong correlation between the wavelength values. Moreover, the fabricated sensor showed specific detection of sHER2 in the presence of other potential biomarkers, as evident from Figure 8(d).

**Figure 8:**
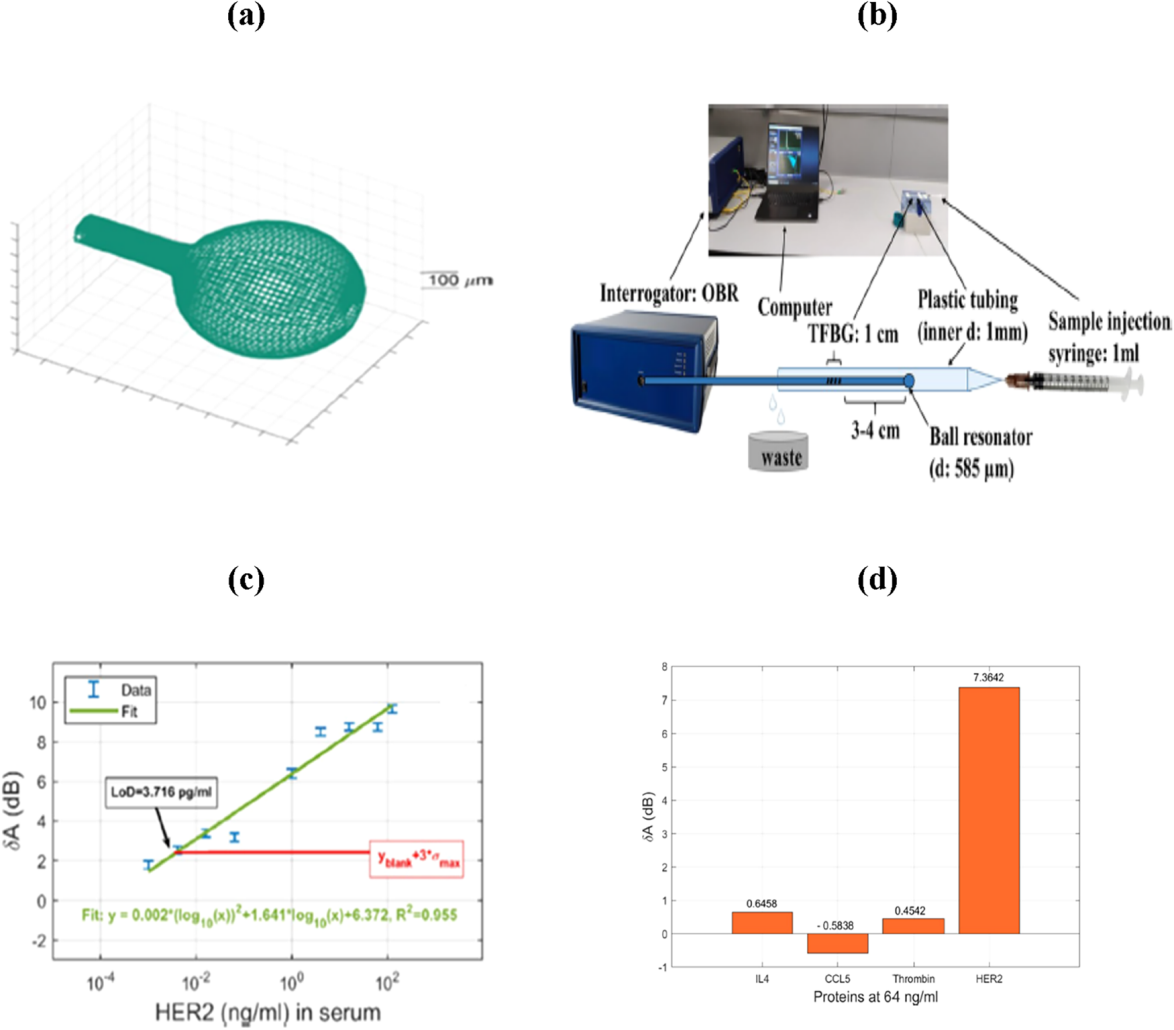
(a) Designed profilometry of ball resonator. (b) Schematic diagram of the sensing setup (Photograph courtesy of Sypabekova, M.; Amantayeva, A.; Vangelista, L.; González-Vila, Á.; Caucheteur, C.; Tosi, D., Copyright 2022, American Chemical Society). (c) Variation of amplitude for different sHER2 concentrations in 1/10 diluted serum. (d) Bar diagram showing the specificity of fabricated sensor toward sHER2 biomarker. Figures reprinted with permission under the terms of CC BY license from ref 180. Copyright 2022, American Chemical Society.

Recent advancements have combined FBGs with PCFs to exploit cladding mode resonances, improving performance for HER2 detection across a wide concentration range.^181^ The sensor used the concept of cladding mode resonances for HER2 sensing, and the corresponding wavelength shifts for these resonances were measured simultaneously for each FBGs. The fabricated sensor was then tested for different concentrations of HER2 in PBS (i.e., 1 ng/mL, 10 ng/mL, 100 ng/mL, and 1 μg/mL) with a total sample volume of 500 μL. Recently, an Au-coated D-shaped optical fiber SPR sensor using antigen–antibody binding scheme was developed to monitor low concentrations of HER2 biomarker.^182^ After modifying the sensing fiber with anti-HER2 antibodies, the sensor showed a wavelength shift of 1.37 nm for 1 μg/mL HER2 concentration, with the corresponding values of LOD and response time being 5.28 nM and 5 s, respectively.

##### 3.2.1.2 Cluster of Differentiation 44 (CD44)

While HER2 is primarily associated with tumor subtype classification and targeted therapeutic stratification, CD44 serves as a surface marker of breast cancer stem cells (CSCs) and is closely linked to tumor initiation, metastasis, and recurrence.^183,184^ Therefore, CD44 provides complementary diagnostic and prognostic information, particularly in assessing tumor aggressiveness and therapeutic resistance. The significant concentration of CD44 in a clinical sample was found to be 400–500 ng/mL. But it is worth noting that detecting these CSCs in breast cancer is forever a massive challenge in clinical trials, as the number of CSCs in actual sample is very low.

Tosi et al. reported an optical fiber ball resonator sensor combined with Karhunen-Loeve transform (KLT) analysis to determine CD44 concentrations.^185^ The Au-coated surface of the ball resonator was functionalized with CD44 antibodies, and an optical backscattering reflectometer was used to sense the CD44 having concentrations ranging from 0.006 to 100 nM. The LOD for the developed sensor turned out to be 19.7 pM, with very good specificity (∼40%). Soon, another OFB having a spherical tip deposited with thin zinc oxide (ZnO) film having thickness 100 nm was designed to detect different CD44 concentrations, i.e., from 100 aM to 100 nM.^186^ For sensor fabrication, the spherical tip fiber surface was washed with Piranha solution before coating the ZnO film and then modified with CD44 antibody. The wavelength regime in which the developed sensor had the most sensitive response was selected for LOD calculation, which turned out to be 2.13 fM. In a similar work, Ashikbayeva et al. described a green-synthesized gold nanoparticles (AuNPs)-based OFB with enhanced sensitivity for CD44 detection.^187^ AuNPs were prepared from green tea leaves and functionalized on the surface of a Au-coated ball resonator followed using 4 μg/mL CD44 antibody. The proposed sensor reported an LOD of 0.111 pM over a wide range of CD44 concentrations in serum (42.0 aM to 100 nM), with a sensitivity of 13.17 dB in intensity changes.

##### 3.2.1.3 Estrogen Receptor (ER)

ERs are nuclear receptors that are stimulated by estrogens after diffusing into the cell membrane and regulate different pathological and physiological activities in the human body.^188^ Detection of ER helps to identify whether breast cancer patients are suitable for endocrine therapy to reduce tumor growth.^189^ ERs are classified into two independent genes based on their location on the chromosomes, ER-α (located on chromosome 6) and ER-β (located on chromosome 14).^190^ It was observed that ER-β was ample in many normal breast cancer epithelial cells and was present in 20–30% of breast cancer patients, and the positive rate was enhanced to 60% over time.^191^

Liu and co-workers developed a reusable OFB that operates through competitive binding between xenoestrogens and 17β-estradiol (E2), producing a fluorescence signal proportional to free E2.^192^ This platform successfully differentiated the relative estrogenic potencies of diethylstilbestrol, 4-n-octylphenol, and 4-n-nonylphenol, and the E2-functionalized fiber surface could be regenerated for over 300 cycles without a significant loss of performance. Building on this, EW-based biosensor was designed using a triple-functional small-molecule protein.^193^ Upon exposure to xenoestrogens, the conjugate was competitively displaced, and its streptavidin moiety binds to a desthiobiotin-modified fiber surface, producing a measurable fluorescence signal. The sensor demonstrated a linear response over 20.8–476.7 μg/L E2 equiv and a detection limit of 1.05 μg/L E2 equiv. Additionally, Hamid et al. demonstrated a TOF biosensor for 17-ethinylestradiol (EE2) detection, where a 12 μm waist fiber fabricated via heat-and-pull enabled EW interaction with immobilized EE2-specific antibodies.^194^ The TOF sensor exhibited a linear response between 1 and 10 ng/L, a detection limit of 1 ng/L, and high selectivity against estrone.

##### 3.2.1.4 BRCA1/BRCA2 Genes

BRCA1 and BRCA2 are crucial human genes that encode tumor suppressor proteins responsible for maintaining genomic stability through DNA repair mechanisms.^195^ Mutations or structural abnormalities in these genes disrupt normal DNA repair pathways, significantly contributing to breast cancer development. Epidemiological studies indicate that women carrying BRCA1 mutations have an estimated 60% lifetime risk of developing breast cancer, while BRCA2 mutations confer a risk of approximately 50%.^196^ Collectively, these genetic alterations account for nearly 20–25% of hereditary breast cancers and 5–10% of all diagnosed breast cancer cases.^197^ To address the need for early and accurate detection of such genetic mutations, a graphene-coated OFB was proposed and theoretically analyzed for the detection of BRCA1 and BRCA2 mutations.^198^ The sensor operates on the principle of DNA hybridization to identify specific mutations—916delTT in BRCA1 and 6174delT in BRCA2—through distinct variations in the SPR angle and surface resonance frequency. The study demonstrated clear discrimination between perfectly matched and mismatched DNA strands, validating the biosensor’s high specificity for genetic recognition. The integration of graphene significantly enhanced sensitivity due to its exceptional adsorption properties and high carrier mobility, which intensified the localized electric field at the Au–graphene interface.

##### 3.2.1.5 Cancer Antigen 15–3 (CA 15–3)

CA 15–3 is a glycoprotein encoded by the mucin-1 (MUC1) gene, which serves as an established serum biomarker for the diagnosis and monitoring of metastatic breast cancer.^199^ In healthy individuals, the normal concentration of CA 15–3 in serum is typically below 30 U/mL;^200^ however, elevated levels have been reported in more than 70% of breast cancer patients.^201^ To enhance the sensitivity and reliability of CA 15–3 detection, a PCF-based SPR biosensor has been theoretically developed.^202^ This design exhibited high wavelength (42,000 nm/RIU) and amplitude sensitivities (105 RIU^–1^), enabling precise detection of subtle RI variations associated with different CA 15–3 concentrations (3 to 32 IU/mL) in serum.

##### 3.2.1.6 Michigan Cancer Foundation 7 (MCF-7)

MCF-7 cell line, derived from a pleural effusion of a late-stage breast cancer patient, has long served as a well-established in vitro model for breast cancer research.^203^ Extensive cytogenetic and molecular studies such that karyotyping, comparative genomic hybridization (CGH), array CGH, single-nucleotide polymorphism arrays, and gene expression profiling have revealed numerous chromosomal alterations in MCF-7 cells, providing crucial insights into their tumorigenic potential and genetic instability.^204^

In 2020, an etched multicore fiber-based LSPR biosensor demonstrated remarkable sensitivity toward MCF-7 detection.^205^ By integration of AuNPs, GO, and copper oxide nanoflowers, the sensor achieved enhanced plasmonic coupling and biocompatibility. Functionalization with 2-deoxy-d-glucose further facilitated specific recognition of MCF-7 cells via glucose transporter-mediated interactions. The sensor achieved an LOD of 2 cells/mL across a linear range of 10^2^–10^6^ cells/mL, along with excellent selectivity and reproducibility. Building upon this, Mollah et al. developed a SI-based PCF sensor for distinguishing MCF-7 cells from normal cells through RI variations.^206^ The introduction of an elliptical core enhanced birefringence and optical confinement, achieving an impressive RI sensitivity of 31,429 nm/RIU. More recently, Sardar and Faisal proposed an SPR-based PCF sensor meticulously optimized via the finite element method (FEM).^207^ Fabricated through sol–gel and stack-and-draw techniques, the structure incorporated strategically arranged air holes to maximize light-matter interaction, while a Au layer deposited by chemical vapor deposition (CVD) boosted its SPR response. The optimized configuration achieved a wavelength sensitivity of 7143 nm/RIU for the MCF-7 cells.

### 3.3 Liver Cancer

Liver cancer ranks as the third leading cause of cancer-related mortality globally and remains one of the most lethal malignancies.^3^ Among its various forms, hepatocellular carcinoma (HCC) represents nearly 90% of all liver cancer cases, and it is primarily associated with chronic viral hepatitis infections, i.e., hepatitis B virus (HBV) and hepatitis C virus (HCV).^208,209^ While widespread HBV vaccination programs and the availability of effective oral antivirals for HCV have significantly reduced the incidence of virus-related HCC worldwide, other risk factors have gained prominence. The rising prevalence of alcohol misuse, obesity, and metabolic syndrome has led to a growing burden of nonviral liver cancers, particularly in high-income regions such as the United States, Europe, and parts of Asia.^210^ These shifting trends highlight the need for integrated prevention strategies to effectively reduce the global incidence of HCC.

#### 3.3.1 Biomarker for Liver Cancer and its Detection

α-fetoprotein (AFP) is a glycoprotein primarily synthesized by the fetal liver and yolk sac, with an approximate molecular weight of 70 kDa.^211^ It serves as a key clinical biomarker for the early detection and diagnosis of HCC.^212^ In healthy adults, AFP levels in the bloodstream are typically below 25 ng/mL. In contrast, patients with HCC often exhibit markedly elevated AFP concentrations, frequently reaching or exceeding 500 ng/mL.^213^ Moreover, monitoring AFP concentrations in serum and urine is of great clinical value for evaluating treatment efficacy, predicting disease recurrence, and assessing overall patient prognosis.^214^

Li et el. developed an optical microfiber (OMF) sensor incorporating AuNPs for the detection of AFP in serum samples.^215^ Theoretical analysis revealed that optimal sensing performance can be achieved when the microfiber diameter is comparable to the operating wavelength, and sensitivity can be further improved by reducing the fiber diameter or increasing the AuNPs size. Experimentally, an OMF of 1 μm diameter was functionalized with 40 nm AuNPs and demonstrated detection limits of 0.2 ng/mL in PBS and 2 ng/mL in bovine serum. In another study, GO was employed as a functional material over OMF sensor due to its abundant oxygen-containing groups and active binding sites.^216^ The proposed biosensor exhibited a sensitivity of 1.11582 nm/log­(mol/L) within a linear detection range of 7 zg/mL to 7 pg/mL and achieved an LOD of 78 zg/mL.

Building on this, Liang and co-workers introduced a U-shaped LSPR sensor for the detection of AFP.^217^ The U-shaped optical fiber was pretreated with microwave-induced H_2_O/Ar plasma-enhanced silanization, which improved AuNPs adhesion and surface uniformity. The developed biosensor exhibited a wide detection range of 5–200 ng/mL with detection limits of 0.85 ng/mL in PBS and 3.3 ng/mL in human serum. In a similar work based on LSPR technique, a slide-type fiber structure was integrated with MMF–multicore fiber–MMF configuration to further enhance light leakage and evanescent field interaction.^218^ The sensor used AuNPs for LSPR generation, cerium oxide (CeO_2_) NPs and carbon quantum dots for amplifying the signal, and AFP-specific antibodies for selective biomolecular recognition. The system achieved a sensitivity of 32 ng/mL ^–1^ and a detection limit of 6.65 ng/mL for rapid and accurate AFP detection. To further improve sensing performance, the fiber surface was functionalized with ZnO nanowires, tungsten disulfide nanosheets, and AuNPs.^219^ The biosensor exhibited a sensitivity of 1.32 nm/log­(ng/mL) and LOD of 84 pg/mL within a wide detection range from 0 to 1000 ng/mL.

In another study, PCF-based immunosensor reported a detection limit of 0.1 ng/mL within linear range of 0.1–150 ng/mL for the detection of AFP in human serum.^220^ Furthermore, a plasmonic-based OFB was developed by employing dispersion theory and finite element analysis (FEA).^221^ Dispersion modeling based on ray optics provided insight into the structural configuration, while FEA modeling guided the selection of materials through an optimized dielectric constant ratio between the real and imaginary components. Incorporation of a polydopamine (PDA) layer improved antibody immobilization and near-field coupling, leading to a high biosensing efficiency. As shown in Figure 9(a–d), significant shift in RW values can be seen with time for AFP concentrations ranging from 0.1 to 100 ng/mL with both Logistic and Langmuir fitting of data points. This device achieved detection limits of 0.01 ng/mL (logistic fitting) and 0.05 ng/mL (Langmuir fitting), demonstrating excellent reproducibility and accurate AFP detection, as evident from Figure 10(e,f).

**Figure 9:**
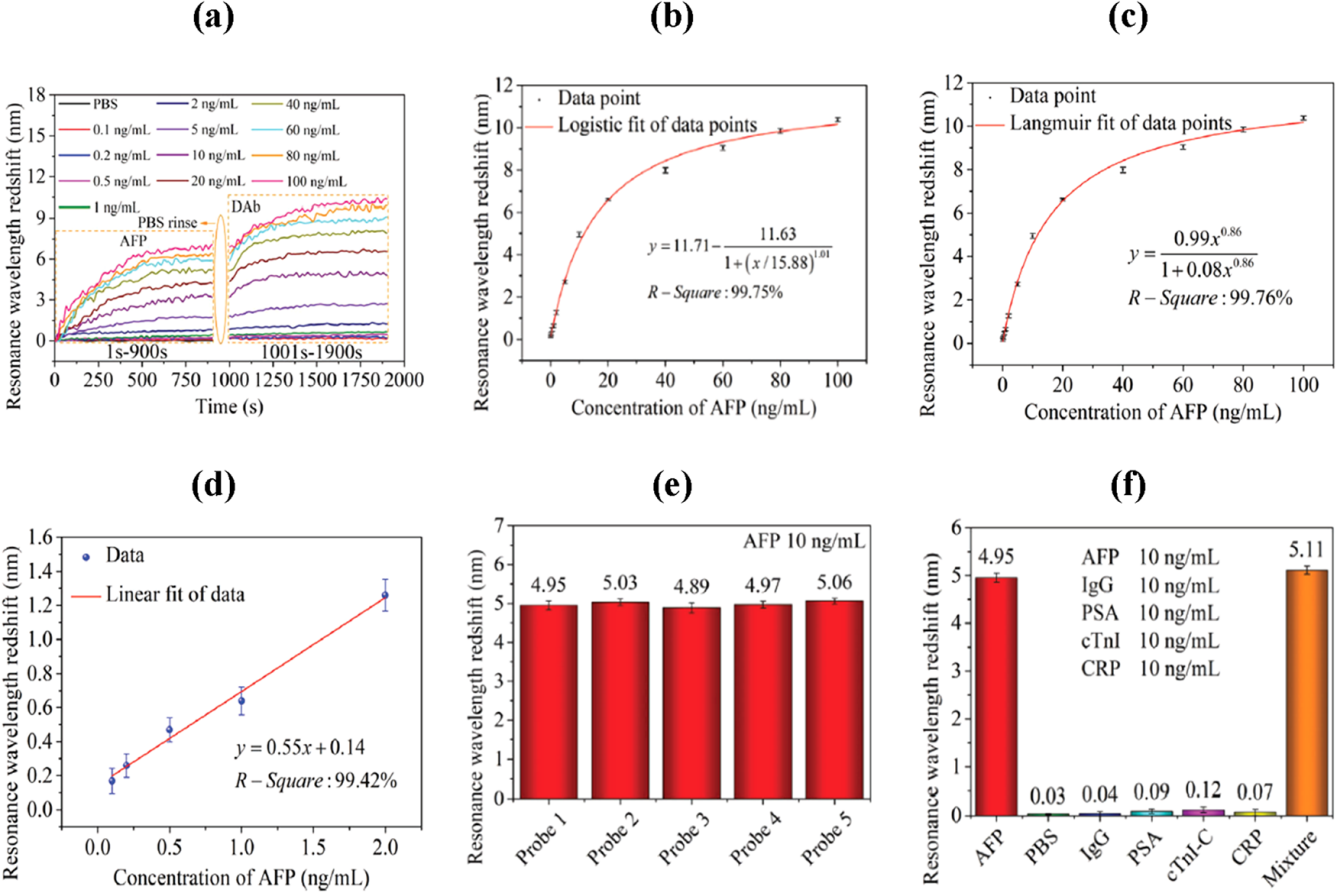
(a) Shift in RW observed over time for the detection of AFP concentrations. (b) Logistic fitting, (c) Langmuir dynamic calibration plots, (d) linear correlation between RW redshift and AFP concentration within the range of 0.1–2 ng mL^–1^, (e) evaluation of the repeatability, and (f) assessment of the specificity of the developed sensor. Figures reprinted with permission under the terms of CC BY license from ref 221. Copyright 2023, Wiley VCH.

**Figure 10:**
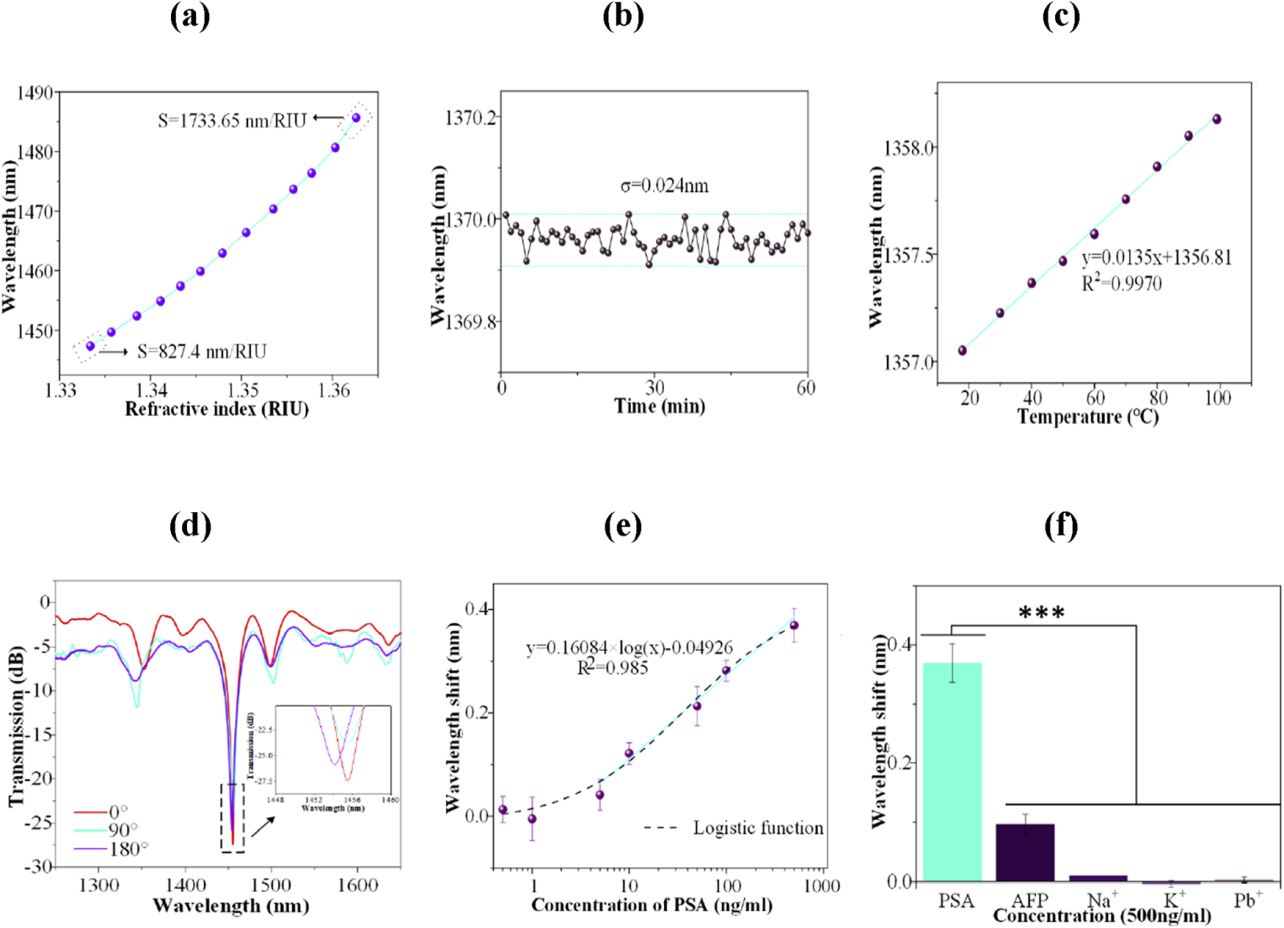
(a) Variation in RW of the HOD-LPG sensor with changes in SMRI. (b) Stability analysis with time, (c) dependence of RW on ambient temperature, (d) transmission curves of the HOD-LPG sensor for various bending curvatures, (e) experimental response of the proposed biosensor as a function of PSA concentration, and (f) specificity analysis. Figures reprinted with permission under the terms of the OSA Open Access Publishing Agreement from ref 240. Copyright 2020, Optical Society of America.

In 2024, an SPR-based reflective OFB was developed for the detection of AFP using a boronate affinity-based MIP approach.^222^ Ag-coated MMFs were functionalized with boric acid, enabling selective recognition of AFP via reversible cis-diol interactions. The sensor exhibited a linear detection range of 0–1 ng/mL, sensitivity of 2.72 nm/log­(pg/mL), good linearity (*R* ^2^ = 0.997), and high stability under varying pH and temperature. Another SPR sensor featuring a chimney-like microstructure was fabricated on the end-face of an SMF with dimensions of 10 μm × 10 μm × 16 μm.^223^ The study also included the finite-difference time-domain (FDTD) simulations, which guided the optimization of geometric parameters. Experimentally, the sensor exhibited an AFP sensitivity of 0.684 nm/(ng/mL), an RI sensitivity of 657.33 nm/RIU, and an LOD of 0.029 ng/mL, along with good selectivity.

### 3.4 Prostate Cancer

Prostate cancer is the second most frequently diagnosed malignancy among men and the fifth leading cause of cancer-related deaths worldwide.^224,225^ By 2024, an estimated 1.9 million new cases were diagnosed, resulting in about 600,000 deaths, which is nearly 5% of all male cancer fatalities.^226^ The global incidence of prostate cancer shows significant geographic variation, with the highest rates reported in North America, Oceania, and Europe, and the lowest in Southeast Asia.^227^ Prostate cancer predominantly affects men between 45 and 60 years of age, though cases have also been documented in individuals under 45.^228^ The major risk factors include age, family history, and genetic predisposition; however, no definitive preventive measures have yet been established.^229^ Common clinical manifestations include difficulty in urination, weak urinary flow, and persistent pain in the lower back, hips, or thighs. A substantial proportion of patients eventually develop resistance to androgen deprivation therapy, progressing to metastatic castration-resistant prostate cancer (mCRPC).^230^

#### 3.4.1 Biomarker for Prostate Cancer and its Detection

The prostate gland secretes a glycoprotein known as prostate-specific antigen (PSA), a biomarker with a molecular weight of approximately 33 kDa that plays a crucial role in the early diagnosis of prostate cancer.^231,232^ PSA is present in various biological fluids, including serum, seminal plasma, benign prostatic hyperplastic fluids, and prostatic secretions.^233^ Clinically, serum PSA concentrations above 10 ng/mL are often indicative of prostate malignancy, whereas levels below 4 ng/mL are typically considered normal.^234^ Moreover, in patients who have undergone prostate cancer surgery, PSA values exceeding 0.5 ng/mL are of significant clinical concern, as they may suggest disease recurrence or progression.^235^

Early developments include LSPR-based OFB with Au nanodisk arrays on the fiber tip, functionalized with anti-PSA monoclonal antibodies.^236^ Specific detection was achieved through the immobilization of mouse antihuman PSA monoclonal antibodies on a self-assembled monolayer (SAM) at the LSPR-active fiber interface. The sensor achieved RI sensitivity of around 226 nm/RIU and LOD of 3 fM, within PSA detection range of 100 fg/mL to 5 ng/mL, demonstrating high specificity and selectivity in PBS. To overcome limitations such as air exposure and NPs instability, LSPR-based OFBs were integrated with microfluidic channels, allowing continuous fluid delivery to the sensor surface.^237^ This approach mitigated variability in NPs size and distribution, enabling sensitive PSA detection with a LOD of 124 fg/mL and a dynamic range spanning 4 orders of magnitude. The sensor exhibited good linearity, specificity, and accuracy, including successful detection in patient serum samples. Sensor performance was further enhanced by using 3D LSPR-OFB architectures. For instance, incorporating ZnO nanowires with AuNPs on the fiber tip increased the sensing area and improved analyte accessibility.^238^ Compared to conventional 2D LSPR-OFB, the 3D structures increased the RI sensitivity by 171% and reduced the PSA detection limit to 0.51 pg/mL, representing a 404% improvement. Also, to improve reproducibility and stability, a focused ion beam (FIB) milling was employed to pattern nanostructures directly from metallic films on optical fibers.^239^ FIB-fabricated LSPR-OFB achieved a PSA detection limit of 0.1 pg/mL with reproducible results.

High-order-diffraction long-period gratings (HOD-LPGs) have also been developed for PSA detection, offering temperature-insensitive and bending-independent performances.^240^ The HOD-LPG response was studied for RIs ranging from 1.333 to 1.365, showing sensitivities of 827.4 nm/RIU at 1.3334 and 1733.65 nm/RIU at 1.3626 (Figure 10a). Excellent stability was observed, with a wavelength fluctuation of only 0.024 nm (Figure 10b). Moreover, the sensor’s temperature response was recorded from 18 to 100 °C, as depicted in Figure 10(c), and its bending behavior under different curvatures is illustrated in Figure 10(d). In addition, plotted in Figure 10(e,f), the biofunctionalized HOD-LPG microfibers showed selective PSA detection having concentrations ranging from 0.5 to 500 ng/mL with a log–linear response, LOD of 9.9 ng/mL, and a sensitivity of 0.16084 nm/(ng/mL).

After this, Zhao et al. developed an aptamer-based OFB for highly sensitive and efficient detection of prostate cancer.^241^ Target-specific aptamers bridge antibody–antigen interactions on the fiber surface with RCA and streptavidin–biotinylated horseradish peroxidase nanocomposites, generating hundreds of binding sites. Using PSA as a model biomarker, this sensor achieved a detection limit of 3.2 fg/mL over a wide linear range of 0.01–1000 pg/mL, with good reproducibility and accuracy in human serum. Soon after this, a novel peptide-based OFB was developed for the monitoring of PSA in biological samples.^242^ The sensor employed biotinylated peptides immobilized via streptavidin–biotin interactions and horseradish-peroxidase-modified AuNPs as chemiluminescent labels. The proposed sensor exhibited PSA detection from 0.80 to 100 ng/mL, with an LOD of 0.30 pg/mL.

Recent innovations in multiplexed and dual-resonance OFBs have further advanced PSA detection. For instance, a multiplexed dual OMF sensor was developed for sensitive and specific PSA detection.^243^ Functionalized with AuNRs and Au-nanobipyramids to exploit strong LSPR effects, the sensor achieved an LOD of 3.97 × 10^–15^ mol/L. The observed results also demonstrated specificity and successfully quantified PSA in undiluted serum and urine. Similarly, the fabrication of dual-resonance optical fiber LMR immunoprobes combining MgF_2_ layer with ITO was proposed to enhance phase matching and reduce RW cross-talk.^244^ This dual-resonance design improved detection precision, achieving an LOD of 52 pg/mL for TM-polarization and 91 pg/mL for TE-polarization.

### 3.5 Oral Cancer

Oral cancer is recognized as the sixth most common malignancy worldwide, contributing to roughly 300,000 new cases and about 145,000 deaths each year.^3,245^ Among the various types, oral squamous cell carcinoma (OSCC) represents the predominant form and remains a serious global health issue. Its occurrence is notably higher in regions such as Sri Lanka, India, Pakistan, Bangladesh, Hungary, and France.^3,246^ The major etiological factors linked to OSCC include habitual betel quid chewing, tobacco consumption, and heavy alcohol intake.^247^ OSCC is often detected at a late stage, which significantly reduces the five-year survival rate to below 50%.^248^ The disease most frequently arises in the tongue, particularly along its ventrolateral border, accounting for around 40% of cases. The floor of the mouth is the next most common site (about 30%), followed by the lower lip.^245^ Therefore, the precise staging of the disease before initiating therapy is vital for effective management.

#### 3.5.1 Biomarkers for Oral Cancer and Their Detection

Saliva-based biomarkers are increasingly being recognized as a promising and noninvasive tool for the early diagnosis and monitoring of OSCC.^249^ Saliva serves as a dynamic biological fluid rich in proteins, nucleic acids, metabolites, enzymes, and cytokines, all of which reflect both localized oral changes and broader systemic alterations in the body.^250^ Compared to conventional diagnostic fluids such as blood, saliva offers several advantages, such as easy collection, being inexpensive, requiring minimal handling, and being stored without the risk of clotting. Recent developments in the field of salivaomics have facilitated the identification of multiple molecular biomarkers, notably interleukin-6 (IL-6), interleukin-8 (IL-8), and tumor necrosis factor-α (TNF-α), which are often found at elevated levels in individuals with OSCC. The next subsections present a detailed discussion of these biomarker categories, detection schemes, and their integration into OFBs for oral cancer diagnosis.

##### 3.5.1.1 Interleukin-6 (IL-6)

IL-6 is a multifunctional cytokine encoded by the IL6 gene and plays a central role in regulating immune responses, inflammation, and cellular growth.^251^ Although IL-6 is normally produced at very low concentrations in healthy tissues, its levels increase significantly during inflammation and malignancy. In healthy individuals, salivary IL-6 concentrations are typically below 20 pg/mL, whereas patients with OSCC exhibit markedly elevated levels ranging from 0.707 to 435 pg/mL.^252^ This pronounced elevation has made IL-6 a promising biomarker for early-stage cancer detection and disease monitoring. Mechanistically, IL-6 contributes to tumor progression by promoting chronic inflammation, facilitating monocyte recruitment, and influencing DNA methylation patterns that can silence tumor suppressor genes.

One of the recent works reported a silica optical fiber modified with AuNPs and IL-6 capture antibodies for monitoring IL-6 concentrations.^253^ The device demonstrated a detection limit of 1 pg/mL within a minimal sample volume of 1 μL, a linear detection range of 1–400 pg/mL, and spatial resolution between 200 and 450 μm. Another work effectively demonstrated an FBG-based OFB with a semidistributed interferometer for the detection of IL-6 in artificial saliva.^254^ The designed sensor achieved an LOD of 480 aM for IL-6 having concentration ranging from 10 aM to 100 nM for IL-6 and demonstrated specificity against nontarget proteins such as CD44 and TNF-α.

##### 3.5.1.2 Interleukin-8 (IL-8)

IL-8 has a molecular weight of approximately 6–8 kDa, and in physiological conditions, it typically ranges from 52 to 1578 pg/mL in normal tissues. However, in patients with OSCC, its concentration increases dramatically, with reported values lying between 283 and 4082 pg/mL.^252^ Elevated IL-8 levels in saliva have been consistently associated with the presence and progression of oral cancer, making it a valuable biomarker.

In this context, the surface of an OFB was functionalized with anti-IL-8 antibodies for selective detection of IL-8 in artificial saliva.^255^ The fabricated sensor achieved detection limits down to 0.91 fM across a broad concentration range from 273 aM to 59 fM. The sensor performance for IL-8 detection was further improved by combining a fiber optic LSPR sensor with 3D micropillar architectures.^256^ In this approach, micropillar arrays were fabricated on the fiber facet by using single-mask imprint lithography and subsequently functionalized with AuNPs. The corresponding fabrication steps have been shown in Figure 11(a–d). Compared to conventional flat polymer-coated fibers, the 3D micropillars enhanced the effective sensing surface area and improved light trapping through antireflective interface conditions. The sensor resulted in an RI sensitivity of 4.54 and a coefficient of determination (*R* ^2^) of 0.984, as illustrated in Figure 11(e,f). Moreover, Figure 11(g) presents the sensogram measured at 100 pg/mL, depicting the signal variation observed throughout the immunoassay. Using IL-8 as a model biomarker, the LSPR-based OFB enabled quantitative detection across a broad dynamic range (0.1–1000 pg/mL) with a detection limit of 0.013 pg/mL, while maintaining specificity against nontarget proteins (Figure 11h). The combination of enhanced surface area, optimized light-matter interaction, and reproducible NPs functionalization positions this micropillar-based OFB sensor as a robust and portable sensing platform for OSCC diagnosis.

**Figure 11:**
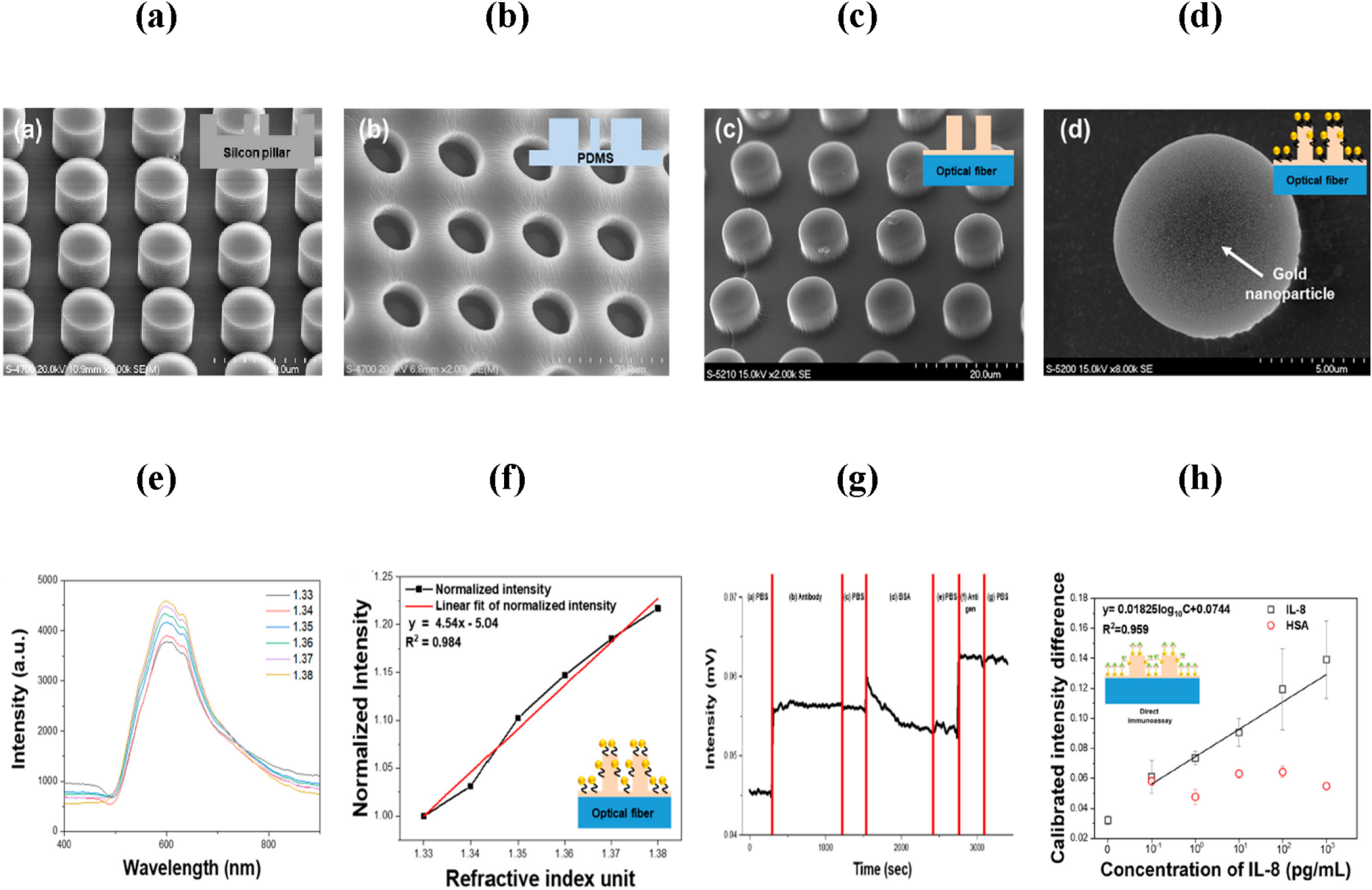
SEM images showing the fabrication steps of the optical fiber LSPR sensor: (a) silicon micropillar array as the primary mold, (b) PDMS secondary mold, (c) polymer micropillars formed over fiber surface, (d) AuNPs deposited on the micropillars, (e) variation of intensity with RIs varied from 1.33 to 1.38, (f) calibration curve for sensitivity measurements, (g) sensogram measured at 100 pg/mL concentration of IL-8, and (h) direct IL-8 detection using a functionalized sensor, where HSA served as a negative control. Figures reprinted with permission under the terms of CC BY license from ref 256. Copyright 2025, MDPI.

##### 3.5.1.3 Tumor Necrosis Factor-α (TNF-α)

TNF-α is a 17 kDa cytokine that plays a critical role in the early detection and assessment of OSCC.^257^ In healthy individuals, salivary TNF-α levels typically range from approximately 2 to 4.5 pg/mL.^258^ In contrast, patients with OSCC often exhibit significantly higher concentrations of TNF-α, reflecting its involvement in tumor development and progression.

Recently, an OFB was successfully developed for the monitoring of TNF-α, achieving a linear detection range of 12.5–200 pg/mL with an LOD of 12.5 pg/mL.^259^ The designed sensing platform also offered flexibility for multiplex detection of other protein targets by simply modifying the capture and detection antibody pairs. After this, another OFB based on a lateral offset spliced coreless MZI was studied for the sensitive detection of TNF-α.^260^ The sensor surface was functionalized with the hydrophobin Grifola frondosa I (HGFI), which enabled SAM formation, facilitating the immobilization of TNF-α antibodies and subsequent specific antigen recognition.

Cao and co-workers have developed a series of high-sensitivity OFBs for the detection of TNF-α, demonstrating progressive improvements in sensitivity and resolution.^261,262^ In 2024, a U-shape single-mode-tapered four-core single-mode (STFS) OFB was fabricated, with its surface functionalized using TNF-α antibodies (10 μg/mL).^261^ This configuration enabled selective antigen binding and exhibited a Hill-type response over a concentration range of 10^1^–10^4^ pg/mL in PBS, achieving the lowest detectable concentration of 10 pg/mL and a wavelength shift of 0.389 nm. This interferometric sensor exhibited an average wavelength fluctuation of 0.099 nm, with corresponding RI sensitivity 1967.24 nm/RIU and LOD 4.35 pg/mL. Application in serum samples confirmed the sensor’s reliability, showing deviations of −0.7% to 14.7% compared with standard TNF-α concentrations. Building on this design, another study integrated an Erbium-doped fiber laser (EDFL) with a single-mode tapered quad-core single-mode (STQS) fiber, further enhancing sensor resolution by reducing the full-width at half-maximum (FWHM) of the detection spectrum from 2.85 to 0.08 nm.^262^ While maintaining a similar detection range and minimum detectable concentration of 10 pg/mL, the EDFL-integrated sensor achieved an increased average wavelength shift of 0.59 nm and excellent stability in PBS, with fluctuations of ±0.12 nm over 30 min. Compared to the initial STFS design, the EDFL-STQS sensor improved sensitivity to 2317.7 nm/RIU with a corresponding LOD of 2.7 pg/mL. These sequential studies illustrate systematic progression in sensor architecture, moving from enhanced interferometric sensitivity (STFS) to improved spectral resolution via EDFL integration and finally to microfiber-based optimization, achieving superior stability and lower detection limits.

In a similar study, single-mode tapered no-core single-mode (STNCS) microfiber biosensor was functionalized with anti-TNF-α antibodies and tested for 1 pg/mL −1 ng/mL concentrations.^263^ Experimental results demonstrated that the STNCS microfiber biosensor exhibits high spectral stability (average wavelength fluctuation of 0.0316 nm), response time of less than 20 min, sensitivity of 2039 nm/RIU, and an LOD close to 1 pg/mL. Specificity tests further confirmed minimal cross-reactivity with other proteins, including C-reactive protein, IL-6, and bovine serum albumin.

### 3.6 Ovarian Cancer

Ovarian cancer is the deadliest form of gynecologic malignancy, ranking eighth globally in both incidence and mortality, with an estimated 295,000 new cases reported each year.^164^ Its prognosis is highly stage-dependent: women diagnosed at early stages (I–II) have an excellent 5-year survival rate (around 90%), whereas survival falls sharply to approximately 20–40% for advanced disease (stages III–IV).^264^ Unfortunately, most patients are diagnosed at a late stage, because early disease produces few or nonspecific symptoms (e.g., persistent bloating, pelvic or abdominal discomfort, early satiety, and urinary urgency).^265^ Consequently, only a minority of cases are detected, while still confined to the ovary, which contributes to the high mortality. Known risk factors include increasing age, genetic susceptibility, obesity, menopausal status, and hormone-replacement therapy, as well as environmental and lifestyle contributors (e.g., smoking, diet, and reduced physical activity).^266^

#### 3.6.1 Biomarkers for Ovarian Cancer and Their Detection

Key biomarkers that have shown significant clinical importance in ovarian cancer include human chorionic gonadotropin (hCG) and cancer antigen 125 (CA-125).

##### 3.6.1.1 Human Chorionic Gonadotropin (hCG)

hCG is a glycoprotein hormone of approximately 36 kDa composed of α and β subunits linked by noncovalent interactions.^267^ Abnormal expression of hCG, particularly its β-subunit, has been closely associated with ovarian cancer.^268^ In healthy women, serum hCG levels are typically below 5 mIU/mL, rising slightly in postmenopausal women up to 14 mIU/mL. In ovarian cancer, β-hCG levels are often elevated, especially in germ cell or choriocarcinomatous tumors, ranging from tens to several thousand mIU/mL depending on tumor type and stage.^269^ Elevated β-hCG levels are frequently detected in the serum of ovarian cancer patients and are considered important diagnostic and prognostic biomarker.

One approach to enhance the intrinsic sensitivity of OFBs for hCG detection was explored through structural optimization and signal amplification. For example, Kumar et al. demonstrated a STNCS fiber sensor with an 8 μm waist and functionalized with magnetic microspheres and dual antibodies (α and β).^270^ This strategy achieved a low LOD of 0.0001 mIU/mL for hCG monitoring with a reduced dynamic measurement range. The work also showed the trade-off between extreme sensitivity and the measurement range. In parallel, microfiber and tapered HCF structures have also been extensively explored for label-free hCG detection. For instance, Chen et al. proposed a single-mode tapered hollow-core single-mode (STHS) fiber sensor with a taper waist of 26.5 μm, showing RI sensitivities of 816, 1601.86, and 4775.5 nm/RIU across different RI ranges.^271^ The study also revealed a clear dependence of thermal and mechanical responses on the taper waist diameter: smaller diameters led to higher temperature and strain sensitivities, with measured values of 26.82 pm/°C (21.4 μm waist) and 7.62 pm/με (20.3 μm waist), respectively. Functionalization with hCG-β monoclonal antibodies enabled detection of 5 mIU/mL hCG concentration with a wavelength shift of 0.5 nm and an LOD of 0.6 mIU/mL. In a similar work, an asymmetrical tapered single-mode multimode single-mode (SMS) coupler comprising two parallel physical-contact SMS was explored.^272^ This design generated two distinct resonance dips sensitive to RI and temperature, allowing for simultaneous measurement of both parameters via matrix inversion. The sensor demonstrated temperature sensitivities of 0.0498 and 0.0324 nm/°C and RI sensitivities of 1151.76 and 1325.66 nm/RIU. Further functionalization enabled detection of 0.05 mIU/mL hCG concentration, achieving wavelength shifts of 0.2 nm. After this, Luo et al. reported a tapered side-polished fiber with a D-shaped section, reduced to <10 μm diameter, and functionalized with primary hCG antibodies.^273^ The sensor exhibited RI sensitivities of 1511.10 nm/RIU and 14219.61 nm/RIU across RI ranges of 1.334–1.349 and 1.402–1.407, respectively, detecting 0.1 mIU/mL hCG with a wavelength shift of 0.82 nm and an LOD of 0.058 mIU/mL. In another study, TOF with waist diameter 3 μm was functionalized with 100 nm AuNPs using the drop-casting method.^274^ The sensor exhibited a strong inverse correlation between transmitted optical power and hCG concentration from 1 to 100,000 mIU/mL, with a spectral redshift of 14.2 nm (Figure 12). A similar type of sensor was also used for the sensitive detection of hCG hormone in aqueous and urine samples.^275^ The sensor achieved sensitivities of 0.012 nm/(mIU/mL) in water and 0.021 nm/(mIU/mL) in urine, with corresponding LODs of 0.04 and 0.03 mIU/mL, respectively. The designed biosensor also showed excellent specificity, as control tests using pure water and urine without hCG produced no detectable spectral shifts. After this, a suspended core fiber spliced between tapered SMFs was employed, where the multimode core and air holes served as microchannels for enhanced light–analyte interaction.^276^ Functionalization with hCG-β antibodies (50 μg/mL) enabled selective detection of hCG with an LOD of 0.049 mIU/mL and RI sensitivity of 1848 nm/RIU. This sensor, based on microchannels, provided an excellent fast response (5 min), anti-interference performance, and stability for hCG detection.

**Figure 12:**
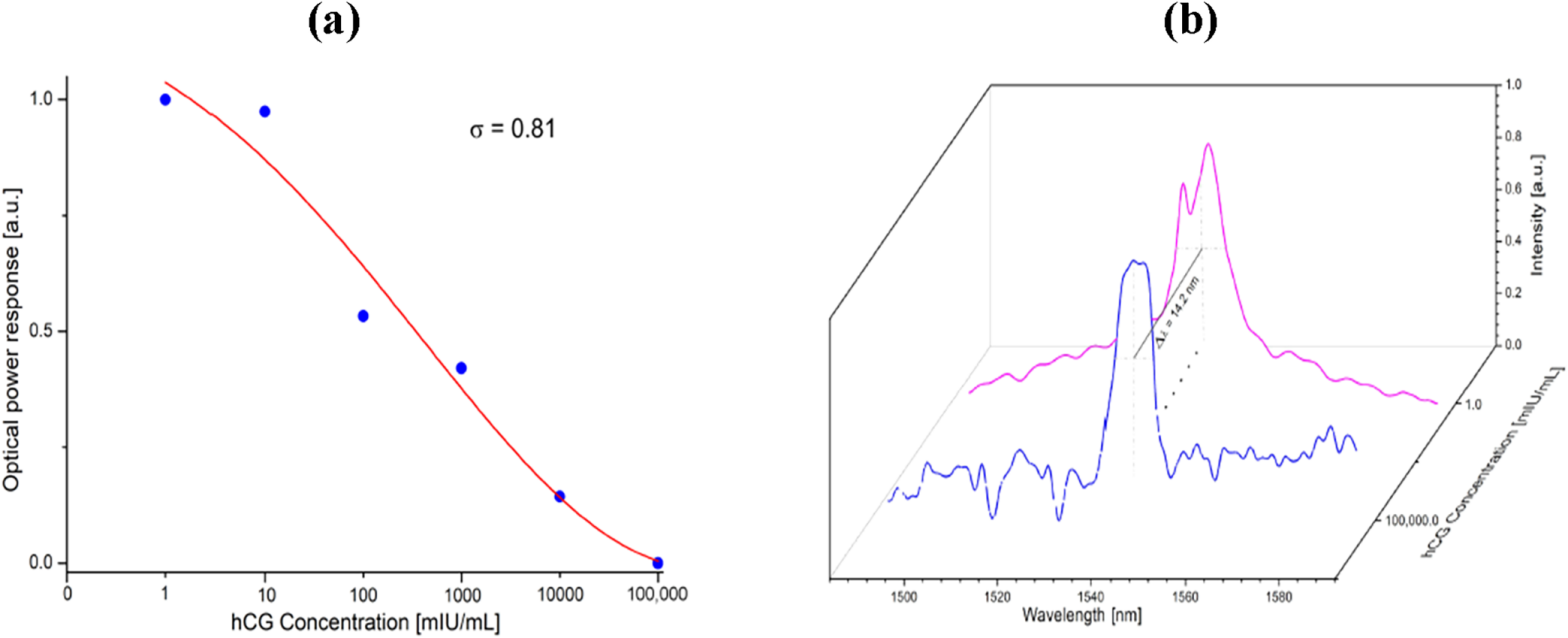
(a) Changes in optical power with increasing hCG concentration (b) corresponding to wavelength shifts. Figures reprinted with permission under the terms of CC BY license from ref 274. Copyright 2023, MDPI.

Finally, temperature-compensated dual-mode OFB represents the latest advancement in high-performance hCG detection.^277^ In this work, a hybrid platform integrating PDA nanostructures with PDMS-modified microcavities, merging SPR and FPI, was developed. PDA facilitated selective antibody immobilization, while PDMS compensated for thermal drift. The sensor achieved dual RI sensitivity of 1875 nm/RIU and differentiated temperature responses (−0.12 nm/°C for SPR and −0.61 nm/°C for FPI). Furthermore, the hCG detection sensitivity reached up to 1.54 nm/(mIU/mL) with an LOD of 0.01 mIU/mL.

##### 3.6.1.2 Cancer Antigen 125 (CA-125)

CA-125 is another established serum biomarker for ovarian cancer diagnosis and monitoring. It is a high-molecular-weight mucin-type glycoprotein commonly overexpressed and secreted by epithelial ovarian tumors.^278^ Normally, CA-125 levels remain below 35 U/mL, but increased concentrations often indicate tumor presence, disease progression, or recurrence.^279^ To sense the CA-125 biomarker, a cost-effective and highly sensitive OFB was developed utilizing the HVE.^280^ The sensor was created by cascading three SMF segments with lateral offsets, forming a cascaded FPI. Experiments revealed RI sensitivities of −5236 and −1163 nm/RIU for the inner and outer interferometric envelopes, respectively. For selective CA-125 detection, the fiber was further functionalized with 50 μg/mL monoclonal antibodies, achieving an LOD of 44.12 fg/mL and a rapid response time of under 30 min.

### 3.7 Thyroid Cancer

Thyroid cancer is the most prevalent malignancy of the endocrine system, ranking ninth among all cancers and seventh among cancers affecting women.^281^ It is most frequently diagnosed in women aged 40–50 and in men aged 60–70, with an average onset age of approximately 51 years.^282^ Women are nearly three times more likely to develop thyroid cancer than men.^283^ Globally, the number of cases has risen sharply from about 238,000 in 2016 to nearly 567,000 in 2018.^284^ Major risk factors include exposure to ionizing radiation, iodine deficiency, family history of thyroid disease, and the presence of goiter or thyroid nodules.^285^ The thyroid gland, located at the base of the neck, produces hormones that are essential for regulating metabolism, growth, energy balance, and cardiovascular function. Thyroid cancer is classified into four major types: papillary, follicular, medullary, and anaplastic.^286^ Papillary carcinoma is the most common form (∼80%), grows slowly, and is highly curable. Follicular carcinoma (∼15%) may spread to bones and distant organs. Medullary carcinoma (∼2%) often arises from inherited genetic mutations, while anaplastic carcinoma is rare but extremely aggressive. Although thyroid cancer is generally treatable, especially when detected early. But its asymptomatic nature in the initial stages often delays diagnosis, underscoring the importance of its early screening to improve survival outcomes.

#### 3.7.1 Biomarker for Thyroid Cancer and its Detection

Thyroglobulin (Tg) is a high-molecular-weight glycoprotein (approximately 660 kDa), produced and secreted by follicular cells in the thyroid gland.^287^ It plays a vital role in thyroid hormone synthesis and serves as a key indicator for detecting and monitoring thyroid malignancies.^288^ In healthy individuals, serum Tg levels are typically below 20 ng/mL, whereas levels above 80 ng/mL may suggest the presence of thyroid cancer.^289^

In this context, Quero et al. utilized a fiber optic nano-optrode sensor based on LPGs for Tg detection.^290^ The sensor, operating in reflection mode, was functionalized with a single layer of atactic polystyrene embedded with high-affinity anti-Tg antibodies. The device exhibited a linear detection range of 0–4 ng/mL and demonstrated 1690 nm/RIU as RI sensitivity. Building on these early developments, Kim and colleagues have systematically advanced plasmonic-based OFBs for the detection of Tg, addressing limitations in sensitivity, detection speed, and reproducibility across a series of studies.^291–296^ In 2019, they developed an LSPR-based OFB using a seed-mediated Au capping process to immobilize AuNPs on optical fibers without binding molecules, enabling controlled NPs size and strong attachment.^291^ This sensor achieved a dynamic detection range from 1 pg/mL to 10 ng/mL and an LOD of 0.19 pg/mL, demonstrating stability through repeated immersion tests. Based on this, authors integrated the LSPR-based OFB with a microfluidic channel to enable rapid Tg detection within 0.001 to 100000 pg/mL, overcoming the slow processing and complexity of conventional assays.^292^ Figure 13(a,b) shows the photograph of the proposed LSPR-based OFB chip and experimental setup used for optical measurements, respectively. The sensor achieved an LOD of 93.11 fg/mL, with measurements completed in ∼10 min.

**Figure 13:**
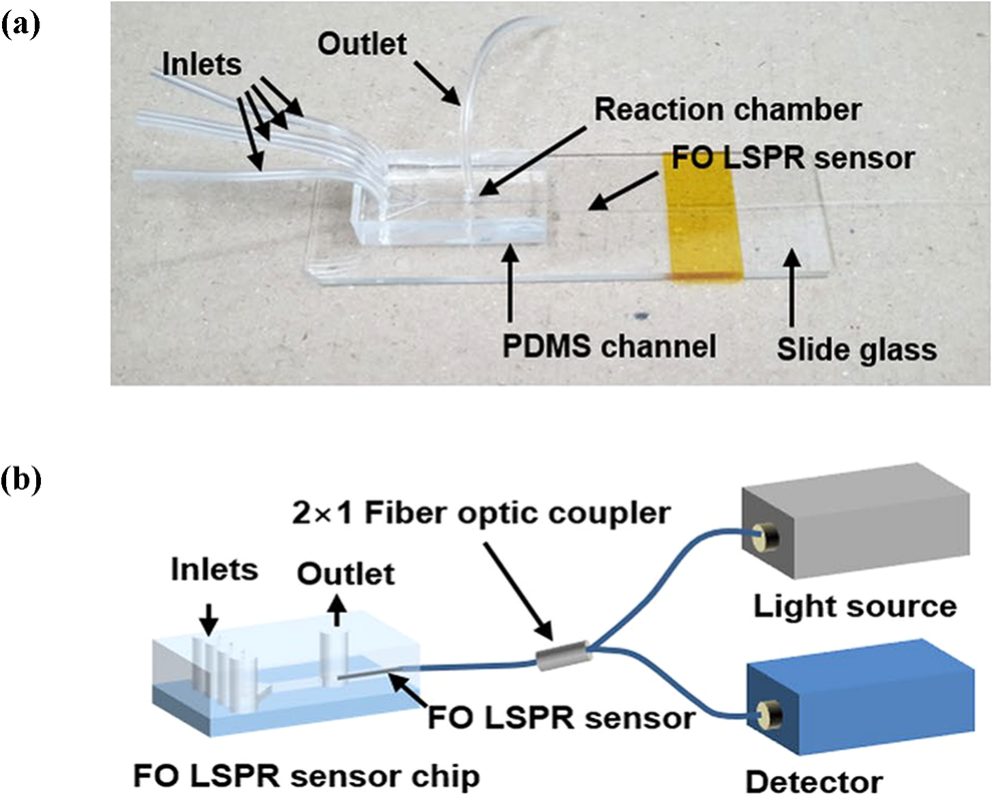
(a) Photograph of the fabricated optical fiber LSPR sensing chip (Photograph courtesy of Kim, H. M.; Jeong, D. H.; Lee, H. Y.; Park, J. H.; Lee, S. K., Copyright 2021, Springer Nature). (b) Experimental setup used for optical measurements. Figures reprinted with permission under the terms of CC BY license from ref 292. Copyright 2021, Springer Nature.

To further enhance sensitivity, a dimer structure of AuNPs on the fiber surface was introduced.^293^ The resulting longitudinal plasmon coupling band increased the sensitivity by 9.1-fold relative to monomer-based sensors, lowering the LOD for Tg and improving assay accuracy. In a follow-up LSPR-based study, multiple fabrication strategies were combined, i.e., optimizing AuNP density, using Au capping to grow particles, and applying reactive-ion etching to reduce background reflection.^294^ This approach further increased the scattering efficiency, decreased background noise, and enhanced reproducibility. Expanding the platform, the authors applied a sandwich immunoassay using secondary antibody functionalization to amplify the plasmonic signal.^295^ This strategy enhanced sensitivity approximately 15-fold and lowered the LOD to 6.6 fg/mL while maintaining selectivity against nontarget antigens. The sensor’s practical applicability was further confirmed by detecting Tg in patient serum. Finally, a high-performance SPR-based OFB incorporating a dome array with nanogaps was developed for 10^–4^ to 10^1^ pg/mL Tg concentrations.^296^ Using polymer beads to form Au nanodomains with nanometer-scale gaps, the sensor created uniform hotspots and expanded the effective sensing area. This nanostructured design enhanced RI sensitivity by 7.8-fold compared to conventional SPR sensors, achieved an LOD of 38 fg/mL for Tg detection, and allowed accurate quantitative analysis in patient serum samples.

Complementing these approaches, Spaziani et al. developed a surface-enhanced Raman spectroscopy (SERS)-assisted immunoassay platform for Tg detection.^297^ The system employed a sandwich immunoassay in which SERS-active substrates were coated with capture antibodies and AuNPs conjugated with detection antibodies and Raman reporters were used for signal enhancement. Fabricated using nanosphere lithography, the fiber-tip-based substrate offered compact and portable configurations. The platform achieved a detection limit of 7 pg/mL and offered high molecular specificity through Raman-based signal discrimination. More recently, an SPR-based OFB was designed by optimizing oriented antibody immobilization, which enabled one-step detection of μg-level Tg concentrations in thyroid tissue fluids.^298^ For nanogram-level detection in serum (0–500 ng/mL), the use of AuNPs-conjugated secondary antibodies provided an approximately 800-fold amplification in signal intensity, leading to a sensitivity of 81.99 pm/(ng/mL) and an LOD of 1.4 ng/mL. The sensor also demonstrated high RI resolution (1.8 × 10^–5^ RIU) and reproducibility with only 1.6% probe-to-probe variation.

### 3.8 Other Types of Cancer and Their Detection

OFBs have also emerged as a promising platform for early diagnosis of other cancers, such as pancreatic, colorectal, skin, cervical, blood, and adrenal gland cancers. For instance, a multiparameter OFB was utilized to detect epidermal growth factor receptor (EGFR) by combining SPR and MZI techniques.^299^ Experimental validation demonstrated a sensitivity of 0.07 nm/nM with an LOD of 3.27 nM. Similarly, a pancreatic cancer biomarker (CA 19–9) was experimentally detected using triangle-based plasmonic nanoprobes and achieved an LOD of 0.25 U/mL.^300^ Recently, PCF-based biosensors have attracted significant attention for the RI sensing of various cancerous cells, including Basal, HeLa, Jurkat, and PC12 cells, corresponding to skin, cervical, blood, and adrenal gland cancers, respectively.^301–304^ These PCF structures enable selective infiltration of biological samples into dedicated cavities, facilitating strong light-matter interactions and highly sensitive detection. For example, a theoretically designed dual-core PCF sensor demonstrated sensitivities of up to 10625 nm/RIU for cervical cancer and 6000 nm/RIU for basal cells under different polarization modes.^305^ Furthermore, a heart-shaped dual-core PCF design was simulated to enhance mode coupling and improve light–sample interaction.^306^ The sensor featured two heart-shaped cores functioning as independent waveguides separated by a large central air hole that served as the sample infiltration region. The FEM was employed to analyze these spectral variations and evaluate sensor performance. This configuration achieved remarkable sensitivities of 7916.67 nm/RIU for cervical cancer, 8571.43 nm/RIU for blood cancer, and 9285.71 nm/RIU for adrenal gland cancer, with a detection limit of 0.024. Advanced PCF geometries, such as ameba-faced designs, have also been developed to further enhance biosensing performance for cancerous cells detection.^307^ Numerically optimized configurations achieved wavelength and transmittance sensitivities over 18,000 nm/RIU and 6000 dB/RIU, respectively. SPR-based PCF sensors have also demonstrated high sensitivity for cancer cell detection. For example, theoretical designs incorporating single or double Au-nanowires have significantly improved coupling between core and plasmonic modes, yielding wavelength and amplitude sensitivity as 2582 RIU^–1^ for PC12 cells.^308^ Similarly, V-shaped PCF sensors coated with zirconium nitride achieved theoretical sensitivity up to 5333 nm/RIU for cervical cancer, with excellent resolution, linearity, and fabrication tolerance.^309^ Recently, graphene–antimonene-coated uniform-waist tapered SPR sensor was simulated, exploiting antimonene high binding energy and large active surface area to enhance the adsorption of cancer biomarkers.^37^ This design achieved sensitivities ranging from 7350 to 15240 nm/RIU for skin, cervical, blood, and adrenal gland cancers, with an LOD of 7.2 × 10^–5^ RIU.

## 4 Summary

This review comprehensively presents recent advancements in OFBs developed over the past decade for the detection of diverse cancer biomarkers. We systematically discussed the major OFB configurations relevant to cancer diagnostics, including resonance-based, grating-based, and interferometric architectures, with concise explanations of their underlying sensing mechanisms. Furthermore, we highlight various cancer types, their implications for human health, risk factors, and the key biomarkers commonly targeted for early detection. Typical concentration ranges of these biomarkers in both healthy and diseased states are also summarized alongside the corresponding OFB-based detection approaches. Table 2 provides a comparative overview of reported OFBs performances in terms of sensitivity, detection range, LOD, and response time for various cancer biomarkers.

**Table 2:** Comparative Analysis of OFBs for Cancer Biomarker Detection and Their Performance Metrics

s. no.	types of cancer	biomarker	sensing configuration	concentration range	sensitivity	LOD	response time	refs
1	Lung	CEA	FPI	500 fg/mL – 5 ng/mL	–8535 nm/RIU	36.14 fg/mL	30 min	^106^
			SPR +TFBG	100 – 1000 ng/mL	0.46 dB (μg/mL)^−1^	505.4 ng/mL	–	^107^
			Interferometric	1 – 50 ng/mL	0.0012 (ng/mL)^−1^	1 ng/mL	–	^108^
			Interferometric	1 pg/mL – 50 ng/mL	1915.9 nm/RIU	15.86 fg/mL	15 min	^109^
		CYFRA 21–1	FPI	0.01 – 100 pg/mL	162000 nm/RIU	1.6 fg/mL	–	^116^
		CK-17	SPR+TFBG	10^–12^ – 10^–6^ g/mL	–	14 fM	–	^114^
			TFBG	10^–12^ – 10^–6^ g/mL	–	14 pM	–	^115^
		CK-7	SPR+TFBG	10^–12^ – 10^–6^ g/mL	–	0.4 nM	–	^113^
		NSE	TFBG	0.01 – 100 pg/mL	1515.3 nm/RIU	1.0 pg/mL	–	^121^
		VEGF	SPR	0 – 100 nM	–	3 nM	–	^125^
		p53	Interferometric	0.05 – 5 ng/mL	3.507 nm/log(ng/mL)	0.0496 ng/mL	–	^129^
		DNA	SPR	0 – 60 nmol/L	0.05 nm/(nmol/L)	9 nmol/L	5 min	^134^
			SPR	0 – 110 nmol/L	0.249 nm/(nmol/L)	–	–	^135^
			SPR	0.07 – 1000 nM	–	88.58 pM	1 s	^137^
		RNA	LSPR	1 pM – 50 nM	–	–	15 min	^138^
		miRNAs	SPR	10^–12^ – 10^–7^ M	–	0.27 pM	–	^139^
			Plasmonic	1 fM – 100 nM	0.98 – 1.01 nm/nM	1.097 – 1.220 fM	–	^143^
			LSPR	10^2^ – 10^7^ fM	–	6.4 fM	–	^147^
			TFBG	10^–18^ – 10^–8^ M	7527 dB/RIU	5.94 – 13.6 aM	–	^149^
		VOCs	LPG	0 – 30 × 10^4^ ppm	0.015 – 0.018 nm/ppm	5.56 – 6.67 ppm	30 s	^154^
			FPI	0 – 70 ppm	281.9 pm/ppm	–	2 s	^156^
			FPI	0 – 400 ppm	–	1 ppb	8 min	^157^
			FPI	0 – 46086 ppm	0.118 – 0.412 pm/ppm	–	185 – 400 s	^158^
			LSPR	0 – 0.39%	15.5 nm/%	0.003%	1.44 – 9.35 min	^159^
			Interferometric	0 – 400 ppm	4.39 – 14.84 pm/ppm	–	9 min	^161^
			SPR	0 – 149 × 10^4^ ppm	–0.525 pm/ppm	–	5 min	^162^
			SPR	0 – 450 ppm	0.014 nm/ppm	82.41 ppm	5 min	^163^
2	Breast	HER2	FBG + Interferometric	0 – 100 ng/mL	2333 nm/RIU	2 ng/mL	–	^174^
			Plasmonic + TFBG	10^–12^ – 10^–6^ g/mL	124.89 nm/RIU	–	–	^176^
			Interferometric	0 – 100 ng/mL	0.1 nm/(ng/mL)	0.5 ng/mL	–	^178^
			SPR	10^–6^ – 10^–12^ g/mL	0.17 nm/nM	9.3 × 10^–9^ g/mL	10 min	^179^
			TFBG	10^–4^ – 10^2^ ng/mL	4034 dB/RIU	3.716 pg/mL	–	^180^
			SPR	0 – 50 μg/mL	28100 nm/RIU	5.28 nM	5 – 30 s	^182^
		CD44	Plasmonic	42.0 aM – 100 nM	13.17 dB	0.111 pM	20 s	^187^
		CA 15–3	SPR	3 – 32 IU/mL	42000 nm/RIU	–	–	^202^
		MCF-7	LSPR	10^2^ – 10^6^ cells/mL	–	2 cells/mL	–	^205^
			SI	–	31429 nm/RIU	–	–	^206^
			SPR	–	7143 nm/RIU	–	–	^207^
3	Liver	AFP	Plasmonic	0 – 1000 ng/mL	–	0.2 – 2 ng/mL	–	^215^
			Interferometric	7 zg/mL – 7 pg/mL	1.11582 nm/log(mol/L)	78 zg/mL	–	^216^
			LSPR	5 – 200 ng/mL	20.25 (AU)/RIU	0.85 – 3.3 ng/mL	–	^217^
			LSPR	0 – 1000 ng/mL	32 pm (ng/mL) ^–1^	6.65 ng/mL	–	^218^
			LSPR	0 – 1000 ng/mL	1.32 nm/log(ng/mL)	84 pg/mL	–	^219^
			Plasmonic	0.1 – 100 ng/mL	0.55[nm/ (ng/mL)]	0.01 – 0.05 ng/mL	–	^221^
			SPR	0 – 1 ng/mL	2.72 nm/log(pg/mL)	–	–	^222^
			SPR	0 – 10 ng/mL	0.684 nm/(ng/mL)	0.029 ng/mL	–	^223^
4	Prostate	PSA	LSPR	100 fg/mL – 5 ng/mL	226 nm/RIU	3 fM	10 min	^236^
			LSPR	1 pg/mL – 10 ng/mL	–	124 fg/mL	–	^237^
			LSPR	0.001 pg/mL – 1 ng/mL	–	0.51 pg/mL	–	^238^
			LSPR	0.1 pg/mL – 1 ng/mL	5700 RIU^–1^	0.1 pg/mL	–	^239^
			LPG	500 – 0.5 ng/mL	0.16084 nm/(ng/mL)	9.9 ng/mL	–	^240^
			LSPR	10^–19^ – 10^–8^ M	0.29191 nm/log_10_ (mol/L)	3.97 × 10^–15^ mol/L	360 – 450 s	^243^
			LMR	0.1 – 20 ng/mL	154.419 – 206.657 nm/RIU	52 – 91 pg/mL	15 min	^244^
5	Oral	IL-6	LSPR	1 – 400 pg/mL	–	1 pg/mL	–	^253^
			FBG+ Interferometric	10 aM – 100 nM	127.3171 dB/RIU	480 aM	–	^254^
		IL-8	LSPR	0.1 – 1000 pg/mL	3.13 nm/RIU	0.013 pg/mL	–	^256^
		TNF-α	Interferometric	10^1^ – 10^4^ pg/mL	1967.24 nm/RIU	4.35 pg/mL	25 min	^261^
			Interferometric	10^1^ – 10^4^ pg/mL	2331.7 nm/RIU	2.75 pg/mL		^262^
			Interferometric	1 pg/mL – 1 ng/mL	2039.952 nm/RIU	1 pg/mL	20 min	^263^
6	Ovarian	hCG	Interferometric	0.05 – 500 mIU/mL	0.25 mIU/mL	0.0001 mIU/mL	–	^270^
			Interferometric	0 – 500 mIU/mL	4540 nm/RIU	0.6 mIU/mL	–	^271^
			Interferometric	0.1 – 100 mIU/mL	1.44 nm/(mIU/mL)	0.058 mIU/mL	–	^273^
			LSPR	1 – 100000 mIU/mL	–	1 mIU/mL	–	^274^
			LSPR	1 – 180 mIU/mL	0.012 – 0.021 nm/(mIU/mL)	0.04 – 0.03 mIU/mL	–	^275^
			Interferometric	0.1 – 100 mIU/mL	1.888 nm/(mIU/mL)	0.049 mIU/mL	5 min	^276^
			SPR+FPI	0.10 – 1000 mIU/mL	1.54 nm/(mIU/mL)	0.01 mIU/mL	–	^277^
		CA-125	FPI	0.0005 – 5 ng/mL	–5236 to – 1163 nm/RIU	44.12 fg/mL	30 min	^280^
7	Thyroid	Tg	LPG	0 – 4 ng/mL	1690 nm/RIU	–	–	^209^
			LSPR	1 pg/mL – 10 ng/mL	13.07 RIU^–1^	0.19 pg/mL	–	^291^
			LSPR	0.001 – 100000 pg/mL	–	93.11 fg/mL	10 min	^292^
			LSPR	0.001 – 10 pg/mL	–	0.050 pg/mL	–	^293^
			LSPR	0.1 – 1000 pg/mL	–	0.14 – 0.5 ng/mL	15 s	^294^
			LSPR	0.997 fg/mL – 9.97 pg/mL	–	6.6 fg/mL	–	^295^
			SPR	10^–4^ – 10^1^ pg/mL	–	38 fg/mL	–	^296^
			SPR	0 – 500 ng/mL	81.99 pm/(ng/mL)	1.4 ng/mL	–	^298^
8	Pancreatic	CA 19–9	LSPR	0 – 37 U/mL	3.32 U/mL	0.25 U/mL	–	^300^

In summary, the past decade has witnessed remarkable progress in both sensitivity enhancement and device miniaturization. Earlier OFB designs were often limited by moderate detection capabilities and relatively bulky interrogation systems. The integration of advanced nanomaterials, optimized fiber geometries, and improved surface functionalization chemistries has progressively reduced detection limits to ultralow concentration regimes suitable for early-stage cancer diagnostics. Simultaneously, advances in microfabrication and compact optical interrogation technologies have enabled the development of highly miniaturized sensing probes and portable configurations.

Beyond improvements in analytical performance, OFBs hold transformative potential in oncology. Their ability to detect low biomarker concentrations using minimally invasive samples supports earlier diagnosis when therapeutic intervention is most effective. The compatibility of optical fibers with multiplexed sensing structures further enables simultaneous detection of multiple biomarkers, improving diagnostic specificity in the context of tumor heterogeneity. Integration with portable instrumentation may facilitate real-time monitoring of disease progression and therapeutic response, contributing to precision oncology strategies.

## 5 Future Prospects

Despite their promising attributes, several scientific and technological challenges still hinder their widespread clinical application. From a biomarker perspective, the primary limitation lies in the biological complexity of cancer, as many biomarkers are not cancer-type-specific and may be expressed in multiple malignancies. This lack of a universal biomarker complicates our understanding of cancer progression and the development of highly selective OFBs. Moreover, the extremely low concentration and nanoscale size of target biomarkers in biological fluids pose additional difficulties in reliable detection.

From an engineering perspective, ensuring fabrication reproducibility, minimizing biofouling in complex matrices such as serum or whole blood, and achieving ultrahigh specificity in multiplexed configurations remain critical challenges across all OFB architectures.^310^ Variations in fiber processing, nanomaterial deposition, and surface functionalization can introduce sensor-to-sensor variability. Nonspecific adsorption may induce signal drift or false-positive responses, while multiplexed formats increase the risk of cross-reactivity and competitive binding effects. Standardized fabrication strategies and robust surface engineering approaches will therefore be essential to improving reliability under clinically relevant conditions.

Looking forward, the next critical phase in the evolution of OFB-based cancer diagnostics lies in rigorous clinical validation and translational development. Although promising proof-of-concept studies and small-scale validations have been reported,^311^ comprehensive evaluation using large and diverse clinical cohorts remains limited. Establishing standardized sensing protocols, ensuring long-term operational stability, and demonstrating reproducibility will be important. In parallel, cost-effective manufacturing strategies and scalable production processes must be developed to support commercialization and broader accessibility.

The integration of artificial intelligence (AI) and machine learning (ML) represents an additional promising direction.^312,313^ As OFB platforms become progressively sensitive and multiplexed, the resulting data sets may exhibit complex and nonlinear patterns that challenge conventional analytical methods. AI-driven signal processing can enhance noise reduction and enable discrimination between specific and nonspecific interactions. ML models trained on large clinical data sets may further support biomarker pattern recognition, risk stratification, and predictive assessment of disease progression.^314^

In addition, certain system-level integration strategies should also be prioritized to bridge laboratory prototypes and deployable diagnostic devices. Coupling OFB platforms with microfluidic modules may enable controlled sample handling, reduced reagent consumption, and automated biomarker capture within compact systems.^315^ Similarly, the development of portable readout units such as miniaturized spectrometers or smartphone-assisted interrogation systems could reduce system footprint and enable point-of-care operation outside centralized laboratory settings.^316^ Such integrated platforms may significantly enhance practical applicability in clinical environments.

## Data Availability

All the data generated/analyzed in this study are included in this article itself.
